# Elucidation of GPR55-Associated Signaling behind THC and LPI Reducing Effects on Ki67-Immunoreactive Nuclei in Patient-Derived Glioblastoma Cells

**DOI:** 10.3390/cells12222646

**Published:** 2023-11-17

**Authors:** Marc Richard Kolbe, Tim Hohmann, Urszula Hohmann, Erik Maronde, Ralph Golbik, Julian Prell, Jörg Illert, Christian Strauss, Faramarz Dehghani

**Affiliations:** 1 Department of Anatomy and Cell Biology, Medical Faculty, Martin Luther University Halle-Wittenberg, Grosse Steinstrasse 52, 06108 Halle (Saale), Germany; marc.kolbe@medizin.uni-halle.de (M.R.K.); tim.hohmann@medizin.uni-halle.de (T.H.); urszula.hohmann@medizin.uni-halle.de (U.H.); 2 Department of Anatomy II, Goethe-University, Theodor-Stern-Kai 7, 60590 Frankfurt am Main, Germany; e.maronde@em.uni-frankfurt.de; 3 Charles Tanford Protein Centre, Martin Luther University Halle-Wittenberg, Kurt-Mothes-Straße 3a, 06120 Halle (Saale), Germany; ralph.golbik@biochemtech.uni-halle.de; 4 Department of Neurosurgery, Medical Faculty, Martin Luther University Halle-Wittenberg, Ernst-Grube-Str. 40, 06120 Halle (Saale), Germany; julian.prell@uk-halle.de (J.P.); joerg.illert@uk-halle.de (J.I.); christian.strauss@uk-halle.de (C.S.)

**Keywords:** calcineurin, endocannabinoid system, Gβγ, IP3-sensitive receptor, NFAT, PLC, ROCK

## Abstract

GPR55 is involved in many physiological and pathological processes. In cancer, GPR55 has been described to show accelerating and decelerating effects in tumor progression resulting from distinct intracellular signaling pathways. GPR55 becomes activated by LPI and various plant-derived, endogenous, and synthetic cannabinoids. Cannabinoids such as THC exerted antitumor effects by inhibiting tumor cell proliferation or inducing apoptosis. Besides its effects through CB_1_ and CB_2_ receptors, THC modulates cellular responses among others via GPR55. Previously, we reported a reduction in Ki67-immunoreactive nuclei of human glioblastoma cells after GPR55 activation in general by THC and in particular by LPI. In the present study, we investigated intracellular mechanisms leading to an altered number of Ki67^+^ nuclei after stimulation of GPR55 by LPI and THC. Pharmacological analyses revealed a strongly involved PLC-IP3 signaling and cell-type-specific differences in Gα-, Gβγ-, RhoA-ROCK, and calcineurin signaling. Furthermore, immunochemical visualization of the calcineurin-dependent transcription factor NFAT revealed an unchanged subcellular localization after THC or LPI treatment. The data underline the cell-type-specific diversity of GPR55-associated signaling pathways in coupling to intracellular G proteins. Furthermore, this diversity might determine the outcome and the individual responsiveness of tumor cells to GPR55 stimulation by cannabin oids.

## 1. Introduction

GPR55 is an orphan G-protein-coupled receptor (GPCR) and has been found within the central nervous system (CNS) in numerous brain regions, such as the hippocampus, striatum, cerebellum, hypothalamus, and cortex, as well as on glial cells and neurons of the dorsal root ganglia [1,2,3,4,5]. GPR55 is also localized outside the CNS in different organs, including the lung, pancreas, liver, spleen, and intestine [1,6,7,8]. Given its ubiquitous distribution, GPR55 is involved in the control of a wide spectrum of physiological processes, including endocrine function, tissue inflammation, and energy metabolism [9]. Recent data have demonstrated that GPR55 is expressed by different human tumor entities, and its activation by its ligand L-α-lysophosphatidylinositol (LPI) has tumor-promoting effects reflected by an increased tumor cell proliferation, migration, and invasion capacity [10,11,12]. 

Glioblastoma, the most common primary brain tumor, is an aggressive and highly invasive tumor characterized by inter- and intratumoral heterogeneity. Despite multimodal treatment strategies, it is considered to remain incurable. It is well discussed that the endocannabinoid system may be a promising target for the treatment of glioblastoma [13,14,15,16,17,18,19]. Exemplarily, phytocannabinoids ∆9-tetrahydrocannabinol (THC) and cannabidiol (CBD) induced cell cycle arrest [18] and apoptosis [20,21] in human glioblastoma cells. Importantly, the complexity of the pharmacology of cannabinoids and their signaling have to be characterized in more detail, allowing the development of targeted and individualized therapeutic interventions [22]. Cannabinoid-receptor 1 (CB_1_) and CB_2_-dependent mechanisms have been reported for anti-tumor activities of THC on glioblastoma cells [14,18,19,20]. However, THC and other CB_1_ and CB_2_ ligands can also modulate cellular responses in a CB_1_/CB_2_-independent manner via other receptors, including GPR55 [15,22]. GPR55 was recently postulated as a novel putative cannabinoid receptor. Among others, plant-derived and endogenous cannabinoids including THC, anandamide (AEA), 2-arachidonoylglycerol, abnormal-CBD, and LPI were found to stimulate GTPγ binding in cells stably expressing GPR55 [1,22]. Furthermore, AEA, an accepted agonist for CB_1_ and CB_2_, exerted anti-proliferative and pro-apoptotic effects on cholangiocarcinoma cells by activating GPR55 [23], although GPR55 is thought to promote tumor cell proliferation of other tumor entities [10,11,12]. These contradictory results might be explained by functional selectivity determined by cell type and ligand utilization as well as by the intracellular events downstream of GPR55 [24].

GPR55 is mainly coupled to Gα_12/13_ [1,2,25,26] or Gα_q_ [2]. Activation of both is associated with stimulation of multiple intracellular signaling pathways, including Ras homolog gene family member A (RhoA) and RhoA-associated protein kinase (RhoA-ROCK) pathway, phosphoinositide 3-kinase (PI3K), and phospholipase C (PLC) [1,2,25,26,27,28]. PLC can be activated directly via Gα_q_ [2] or indirectly via PI3K [29] or RhoA/ROCK [25], resulting in hydrolysis of membrane-bound phosphatidylinositol-4,5-bisphosphate (PIP2) to diacylglycerol (DAG) and inositol triphosphate (IP3). Subsequently, IP3 promotes Ca^2+^ mobilization from endoplasmic reticulum (ER) stores through IP3-sensitive receptors [25,28]. This is followed by an activation of transcription factors by calcineurin, such as nuclear factor of activated T cells (NFAT) [25,30]. In addition, PKC and the downstream RAF/MEK/ERK cascade are activated by DAG and/or Ca^2+^ release, which is accompanied by the recruitment of other transcription factors, such as cAMP response element binding protein (CREB) and nuclear factor kappa light chain enhancers of activated B cells (NF-ĸB) [28,30].

We have recently discovered that THC affected the number of Ki67-immunoreactive nuclei in human patient-derived glioblastoma cells independent of its classical target receptors CB_1_ and CB_2_ [16]. Interestingly, a general activation of GPR55 by its endogenous agonist LPI produced similar effects. Moreover, THC and LPI effects were abolished by specific antagonists [16]. Thus, we concluded that modulating properties of THC on Ki67-immunoreactive nuclei were driven by activation of GPR55 [16]. The present study was designed to shed light on part of the possible intracellular mechanisms downstream of GPR55 that decrease the number of Ki67 immunoreactive nuclei of human glioblastoma when these become activated by THC or LPI.

## 2. Materials and Methods

### 2.1. Cell Culture

Glioblastoma cells designated as *GBM #4* and *GBM #10* were derived from human biopsies as previously described [16]. All patients provided written informed consent. The study was conducted in accordance with the Declaration of Helsinki and was approved by the local Ethics Committee of the University Halle-Wittenberg (project reference number: 2015-144). Some molecular characteristics of the original tumor and cells under investigation are listed in Table S1 [16]. 

Cells were maintained in high-glucose Dulbecco’s Modified Eagle Medium (Invitrogen, Schwerte, Germany, 41965-062) supplemented with 10% (*v*/*v*) fetal bovine serum (FBS, Invitrogen, Schwerte, Germany, 10500-064) and 1% (*v*/*v*) penicillin/streptomycin (Invitrogen, Schwerte, Germany, 15140-122). The passage numbers ranged from 2 to 80.

### 2.2. Treatment

Either 10,000 cells for Ki67 staining or 20,000 cells for NFAT labeling were seeded on sterile coverslips (Dr. Ilona Schubert Laborfachhandel, Leipzig, Germany, 01-0012) in 24-well plates (Greiner Bio-One™, Frickenhausen, Germany, 662160). Cells were allowed to adhere overnight. They were then treated for 24 h with a fresh medium containing THC or LPI (Table 1). Following treatment, the cells were fixed using a 4% (*w*/*v*) paraformaldehyde solution (AppliChem, Darmstadt, Germany, 1.414.511.211). To assess effectors of GPR55-mediated signaling, cells were pre-incubated (marked as ++) with the corresponding inhibitors (Table 1) for varying durations. Subsequently, the medium was replaced by a fresh medium containing THC or LPI with or without inhibitors for 24 h. All treatments were conducted in DMEM supplemented with 10% (*v*/*v*) FBS. Cytotoxic effects of all inhibitors in the absence or presence of THC or LPI were assessed by viability assay (Figures S1 and S2). The concentration of inhibitors was determined based on preliminary experiments in which a range of increasing concentrations of each inhibitor was evaluated (Figures S1–S3). Notably, in cases of high inhibitor concentrations, alternate signaling routes might become activated and induce additional off-target effects.

### 2.3. 3-(4,5-Dimethylthiazol-2-yl)-2,5-Diphenyl-Tetrazolium Bromide (MTT)-Viability-Assay

A total of 3000 cells were placed in 96-well plates (Greiner Bio-One™, Frickenhausen, Germany, 650160) and allowed to adhere overnight. Cells were treated with inhibitors at concentrations specified in Table 1 for 24 h. Four hours before termination of experiments, 3-(4,5-dimethylthiazol-2-yl)-2,5-diphenyltetrazolium bromide solution (MTT, Invitrogen, Schwerte, Germany, M6494) at a concentration of 5 mg/mL was added and incubated for 4 h at 37 °C and 5% (*v*/*v*) CO_2_. After removing the MTT solution, formazan crystals within the cell bodies were dissolved using 100 µL of dimethyl sulfoxide (DMSO, Sigma, Darmstadt, Germany D4540). Absorbance values (Ab) were measured at 540 nm and 720 nm using a microplate reader (SynergyTMMx, BioTek Instruments, Winooski, VT, USA). Control groups consisted of cells incubated in media free of inhibitors, whereas media without cells served as blanks. Cell viability was calculated as follows:

(1) $$viability\left[\%\right]=\frac{{(Ab}_{treated}^{520nm}-{Ab}_{treated}^{720nm})-{(Ab}_{blank}^{520nm}-{Ab}_{blank}^{720nm})}{{(Ab}_{untreated}^{520nm}-{Ab}_{untreated}^{720nm})-{(Ab}_{blank}^{520nm}-{Ab}_{blank}^{720nm})}\times 100$$

All experiments were performed independently at least three times with six technical replicas for each treatment group.

### 2.4. PCR

Total RNA extraction was performed using peqGOLD Trifast™ (Peqlab, Erlangen, Germany, 30-2010). Extracts were treated with a DNA-free™ Kit (Invitrogen, Schwerte, Germany, AM1906) according to the manufacturer’s instructions. RNA concentration was quantified using a Synergy™ Mx Microplate Reader (SynergyTMMx, BioTek Instruments, Winooski, VT, USA). PCR amplification was conducted with 4 µL generated cDNA (Reverse Transcription System, Thermo Fisher Scientific, Waltham, MA, USA, K1691) in 20 µL volume containing 10 µL PCR-MasterMix (Promega Inc., Madison, WI, USA, M7505), 0.5 µL forward and 0.5 µL reverse primer (25 pM), 0.25 µL of EvaGreen dye (Biotium, Fremont, CA, USA, 31000), and 4.75 µL of nuclease-free water (Promega Inc., Madison, WI, USA, P1193) The PCR reaction was performed for 40 cycles using a PCR cycler (7900HT Fast Real-Time PCR System, Applied Biosystems™, Thermo Fisher Scientific, Waltham, MA, USA). Conditions used were as follows: initial denaturation at 95 °C, followed by 40 cycles of denaturation at 94 °C (3 s), annealing at 60 °C, elongation at 72 °C (30 s), and fluorescence detection at 76 °C (*GNA13*, *GNAQ*, *GNAI1*, *GAPDH*, *TPB*) or 80 °C (*GNA12*, *GNAO1*, *GNAI2*, *GNAI3*, *GNASS*, *GNASL*, *POLRR2A*) (15 s). For visualization of PCR products, 30 PCR cycles were performed. Products were separated by electrophoresis on 1.5% (*w*/*v*) agarose gels (PeqLab, Erlangen, Germany, 35-1020) containing GelRed (Biotium, Fremont, CA, USA, 41003) and were visualized using BioTek Synergy Mix (BioTek Instruments, Winooski, VT, USA) (Figure S5). The primers used are listed in Table 2. Relative transcript levels were calculated using the 2^−∆∆Ct^ method. For normalization, RNA polymerase II subunit A (*POLR2A*) served as an internal reference. Normalization to glyceraldehyde-3-phosphate dehydrogenase (*GAPDH*) and TATA-binding protein (*TBP*) were performed to ensure validity and reproducibility as well as to show stable expression of used reference genes in the experimental setup (Figures S6 and S7). 

### 2.5. Immunochemical Staining of Ki67

Endogenous peroxidase activity was blocked by 3% (*v*/*v*) H_2_O_2_ (Roth, Karlsruhe, Germany, 8070.2) in methanol (Roth, Karlsruhe, Germany, AE01.2) for 10 min. Subsequently, cells were washed three times with 0.02 M PBS containing 0.3% (*v*/*v*) Triton X-100 (AppliChem, Darmstadt, Germany, A1388.0500) (PBS/Triton). To minimize unspecific binding, normal goat serum (NGS) (Merck, Darmstadt, Germany, S26) was used at a dilution of 1:20. Cells were incubated overnight at room temperature with primary antibodies (Table 3) appropriately diluted in 0.02 M PBS containing 0.5% (*w*/*v*) bovine serum albumin (BSA) (Sigma, Darmstadt, Germany, A7906) and 0.3% (*v*/*v*) Triton X-100. After rinsing with PBS/Triton, cells were treated with a goat anti rabbit-specific biotinylated secondary antibody for 1 h at room temperature (Table 3). After incubation with ExtrAvidin^®^ peroxidase (Sigma, Darmstadt, Germany, E2886) for 1 h, diaminobenzidine (Sigma, Darmstadt, Germany, D8001) was added together with 0.05% (*v*/*v*) H_2_O_2_ for 3 min. Meyer’s hematoxylin (Hollborn und Söhne GmbH, Leipzig, Germany, H02-0500) was used to counterstain the cell nuclei. Cells were dehydrated in an ascending concentration gradient of ethanol, cleaned with xylene (Roth, Karlsruhe, Germany, 97.13.3), and embedded in Entellan (Merck, Darnmstadt, Germany, 107960).

Staining was visualized by using a Leica DMi8 microscope (Leica, Wetzlar, Germany) at 200× magnification. A total of five different regions on each coverslip were captured. Ki67-positive (Ki67^+^) and hematoxylin-positive cells were counted manually, and the ratios of Ki67^+^ nuclei were determined as previously described [16].

### 2.6. Immunofluorescence Labeling of NFAT

Fluorescence labeling was performed to determine subcellular localization of NFAT isoforms 1–4 after THC or LPI stimulation at different time points (5 min, 10 min, 30 min, 2 h, 4 h, and 24 h). Fixed cells were permeabilized with PBS/Triton for 10 min, followed by incubation with NGS (1:20 in PBS/Triton) for 30 min, before the primary antibody (Table 3) was diluted in 0.5% (*w*/*v*) BSA and PBS/Triton was applied overnight at room temperature. Cells were washed with PBS/Triton and treated for 60 min with Alexa488-labeled secondary antibody (Table 3). Nuclei were counterstained with DAPI (Sigma, Darmstadt, Germany, D5637) for 5 min. Coverslips were mounted on slides with a fluorescent mounting medium (Dako, Jena, Germany, S3023). Images of fixed cells were acquired at 400× magnification using a confocal laser scanning microscope (Leica TCS SPE, Wetzlar, Germany). The following excitation wavelengths were used: 405 nm for DAPI and 488 nm for NFAT isoforms. Emission was detected in the range of Δλ = 415–480 nm (DAPI) and Δλ = 500–570 nm (NFAT).

### 2.7. Statistics

The values were presented as means with standard errors of the mean derived from at least three independent experiments. Because of an uneven number of values, one of the replicas could not be evaluated due to a very low number of assessable cells on the coverslips. All experimental groups were normalized to the untreated control group. Statistical analyses were conducted using either Student’s *t*-test or the one-way ANOVA with Tukey’s post hoc test in GraphPad Prism9 (version. 9.4.1, Boston, MA, USA). The analysis for the normal distribution was performed using the Shapiro–Wilk normality test. All *p*-values referred to the corresponding untreated group or to the group treated with the inhibitor for the relevant effector. Differences were considered significant at *p* ≤ 0.05.

## 3. Results

As LPI, a specific activator of GPR55, reduced the number of Ki67^+^ nuclei via GPR55 to a similar extent as THC under comparable experimental conditions [16], LPI was used as a positive control to demonstrate GPR55-specific signaling. Downstream signaling pathways of GPR55 were investigated after THC or LPI exposure by using inhibitors and antagonists of various intracellular effectors.

### 3.1. Cell-Type-Specific ROCK Signaling and a Strongly Involved PLC-IP3 Signaling

**Figure 1:**
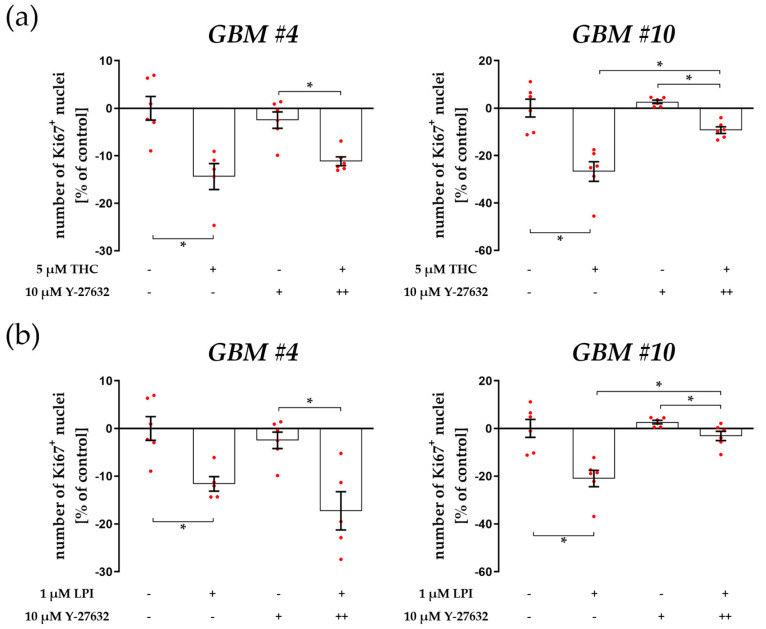
Impact of ROCK inhibitor Y-27632 on THC- and LPI-induced reduction of the number of Ki67^+^ nuclei. *GBM #4* and *GBM #10* were left untreated or exposed to THC (**a**) or LPI (**b**) for 24 h, resulting in a decreased number of Ki67^+^ nuclei. In *GBM #4* THC- (**a**) and LPI (**b**)-mediated effects on the number of Ki67^+^ nuclei remained unaffected in the presence of Y-27632. In contrast, pretreatment with Y-27632 significantly attenuated the responses of *GBM #10* to THC (**a**) and LPI (**b**). Altered numbers of Ki67^+^ nuclei by Y-27632 itself were not observed (**a**,**b**). Data are presented as means ± SEMs of N = 3 independent experiments performed in duplicate. Each red dot represents an individual data point. −/+ indicates without/with the corresponding substance. ++ denotes that cells were pre-incubated with Y-27632 before THC or LPI was added. Significance was set at *p* < 0.05. The asterisk denotes significant results regarding the respective measurement indicated by the bar.

To further confirm GPR55-dependent signaling of THC and LPI, we first evaluated the involvement of known key effectors associated with GPR55 pathways. RhoA and its downstream effector ROCK have recently been implicated as key effectors in GPR55-mediated signaling [25]. As many of the cellular actions of RhoA are mediated by ROCK, the effects of ROCK inhibitor Y-27632 were investigated on THC and LPI responses. In *GBM #10*, the addition of Y-27632 markedly blocked THC- and LPI—mediated changes in the ratio of Ki67^+^ cells (Figure 1a,b, Figures S3a and S4a, Table S2). Interestingly, in *GBM #4*, ROCK was not involved since effects of THC or LPI were still observed in the presence of Y-27632 (Figure 1a,b, Figures S3a and S4a, Table S2). These data indicate a cell-type-specific impact of THC and LPI on the number of Ki67^+^ nuclei via RhoA/ROCK-dependent signaling. 

**Figure 2:**
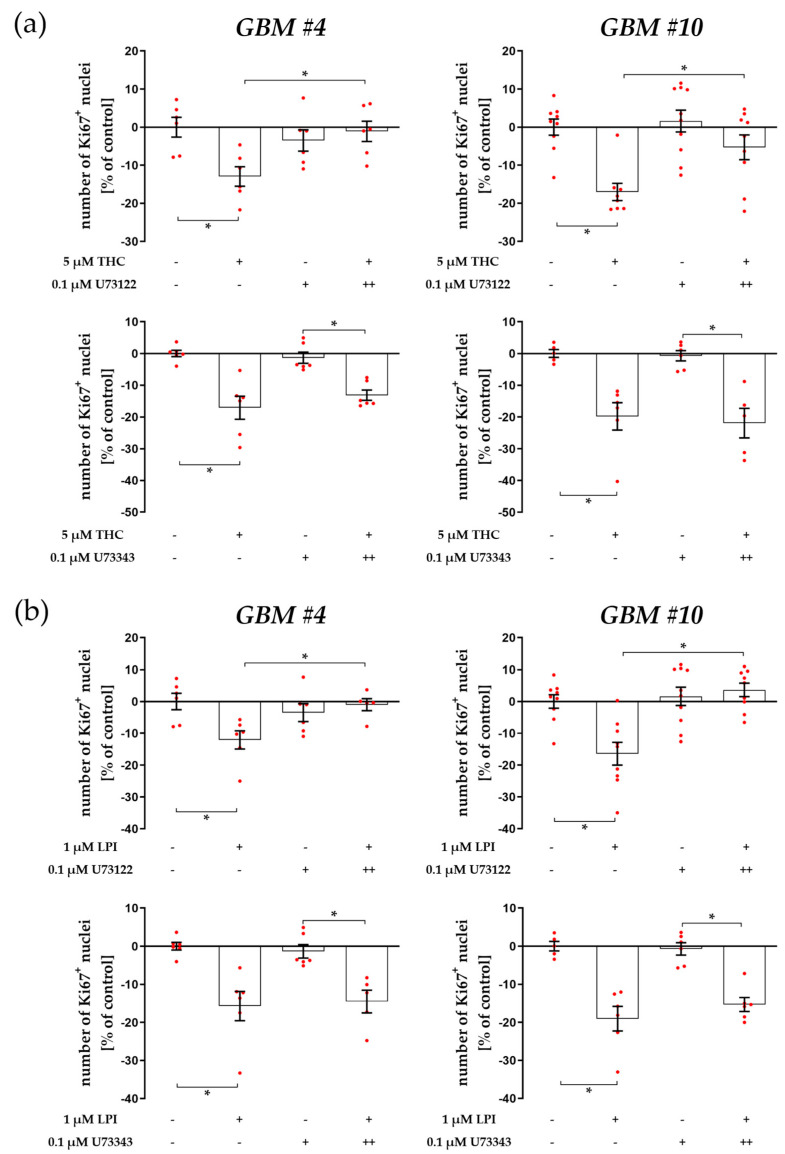
Impact of PLC inhibitor U73122 and its inactive analogue U73343 on THC- and LPI-induced reduction of the number of Ki67^+^ nuclei. *GBM #4* and *GBM #10* were left untreated or exposed to THC (**a**) or LPI (**b**) for 24 h, resulting in a decreased number of Ki67^+^ nuclei. Pretreatment with U73122, a commonly used inhibitor of PLC, significantly reversed the effects obtained after exposure to THC (**a**) or LPI (**b**) in both *GBM #4* and *GBM #10*. Its inactive form U73343 failed to diminish the responses to THC (**a**) and LPI (**b**) at the same concentrations used for U73122. U73122 or U73343 alone did not cause any alterations (**a**,**b**). Data are presented as means ± SEMs of N = 3 independent experiments performed in duplicate. Each red dot represents an individual data point. −/+ indicates without/with the corresponding substance ++ denotes that cells were pre-incubated with U73122 or U73343 before THC or LPI was added. Significance was set at *p* < 0.05. The asterisk denotes significant results regarding the respective measurement indicated by the bar.

PLC has been shown to be another important part of signaling pathways triggered by GPR55 [2,25,36,38]. Thus, cells were treated with PLC inhibitor U73122. U73122 significantly diminished THC effects in *GBM #4* and *GBM #10* (Figure 2a and Figure S3b, Table S3). Similar results were obtained for LPI, as LPI’s effects were no longer detected in the presence of U73122 (Figure 2b and Figure S4b, Table S3). The specificity of U73122 to block PLC was confirmed by using its inactive structural analogue U73343 at the same concentrations used for U73122. Indeed, treatment with U73343 failed to block the responses to THC and LPI in both glioblastoma cell lines (Figure 2a,b, Table S4). U73122 and U73343 themselves displayed no significant effects on the number of Ki67^+^ nuclei (Figure 2a,b, Figures S3b and S4b, Tables S3 and S4). These data indicate that PLC is strongly involved in the regulation of the number of Ki67^+^ nuclei by THC and LPI in both *GBM #4* and *GBM #10*. 

**Figure 3:**
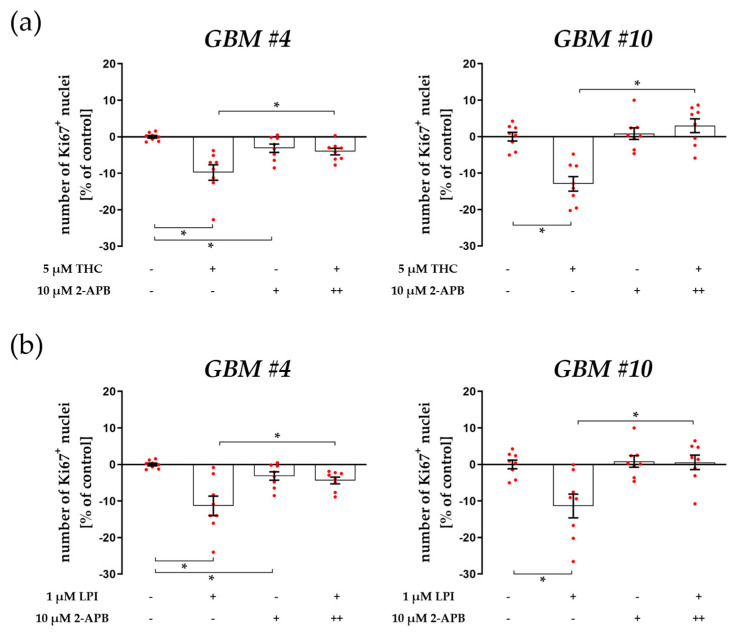
Impact of antagonized IP3-sensitive receptors using 2-APB on THC- and LPI-induced reduction of the number of Ki67^+^ nuclei. *GBM #4* and *GBM #10* were left untreated or exposed to THC (**a**) or LPI (**b**) for 24 h, resulting in a decreased number of Ki67^+^ nuclei. The effects of THC (**a**) and LPI (**b**) were significantly reduced after pre-incubation with 2-APB in both *GBM #4* and *GBM #10*. When *GBM #4* was exposed to 2-APB alone, a small reduction in the number Ki67^+^ nuclei were observed (**a**,**b**). Data are means ± SEMs of N = 4 independent experiments performed in duplicate. Each red dot represents an individual data point. -/+ indicates without/with the corresponding substance. ++ denotes that cells were pre-incubated with 2-APB before THC or LPI was added. Significance was set at *p* < 0.05. The asterisk denotes significant results regarding the respective measurement indicated by the bar.

PLC converts membrane-bound PIP2 to DAG and IP3. Subsequently, IP3 acts as a second messenger and binds to IP3-sensitive receptors on the ER, resulting in an increase in intracellular Ca^2+^ concentrations. To assess whether IP3 and its receptor were involved in the reduction of Ki67^+^ nuclei upon THC or LPI exposure, we used 2-APB to antagonize IP3-sensitive receptors (Figure 3a,b, Figure S3c and S4c, Table S5). Pretreatment with 2-APB attenuated the responses to THC and LPI in both *GBM #4* and *GBM #10* (Figure 3a,b, Figures S3c and S4c, Table S5), making the participation of IP3-mediated signaling downstream of GPR55 evident and further confirming PLC-dependent signaling.

### 3.2. Characterization of Gα- and Gβγ-Subunits, Which Might Couple to GPR55 Signaling

**Figure 4:**
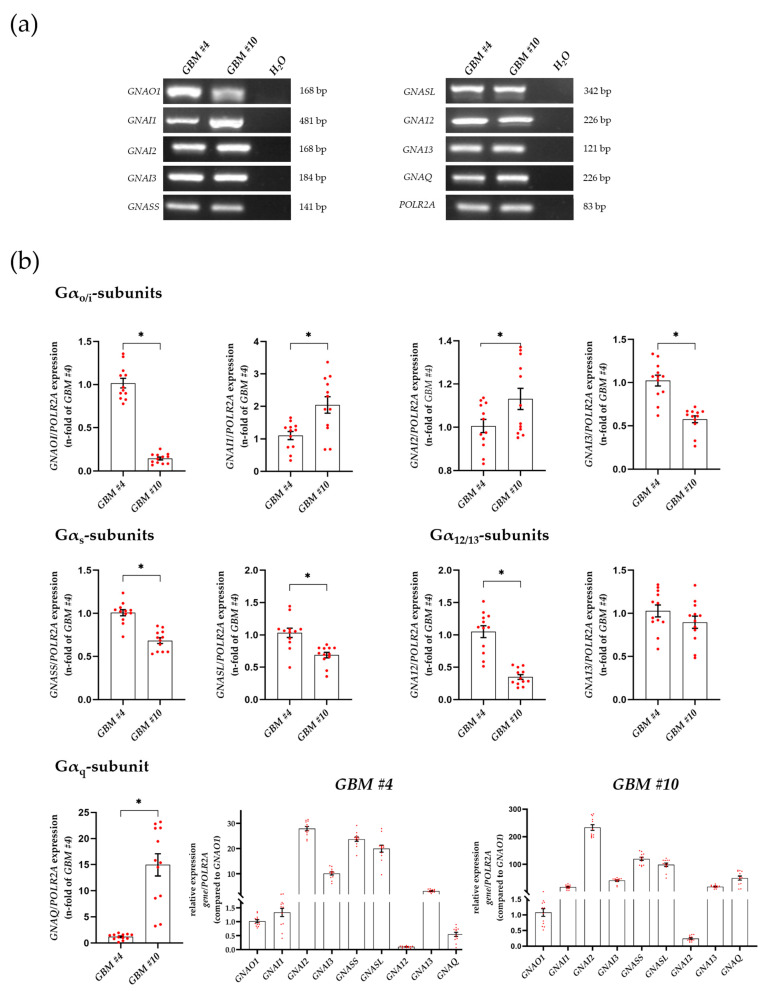
Detection and quantification of genes encoding different Gα subunits at transcript level. Expression of *GNAO1*, *GNAI1*, *GNAI2*, *GNAI3*, *GNASS*, *GNASL*, *GNA12*, *GNA13*, and *GNAQ* were analyzed by quantitative RT-PCR in untreated cells of *GBM #4* and *GBM #10*. All cells expressed the examined Gα-subunits as transcripts (**a**) at different levels (**b**). *RNA polymerase II subunit A* (*POLR2A*) served as an internal reference. Furthermore, relative transcript levels were calculated using the 2^−∆∆Ct^ method (**b**). Remarkably, *GBM #4* showed a significantly higher amount of Gα_o_ transcripts than *GBM #10*, whereas Gα_q_ showed significantly higher expression by *GBM #10* when compared to *GBM #4*. The abundance and distribution of gene transcripts encoding different subunits within one cell population were similar in *GBM #4* and *GBM #10*. Altered ratios to others were observed for *GNAOI* in *GBM #4* and *GNAQ* in *GBM #10*. Data represent means ± SEMs (normalized to *GBM #4* or *GNAOI*) of N = 4 independent experiments performed in triplicate. Each red dot represents an individual data point. Significance was set at *p* < 0.05. The asterisk denotes significant results regarding the respective measurement indicated by the bar.

In light of the diversity of G proteins and their functions, the expression of different G-protein α subunits and their isoforms were examined by qRT-PCR analysis of untreated glioblastoma cells. Our results demonstrated that Gα_o_, Gα_i_ (isoforms 1, 2, and 3), Gα_s_ (small and large isoforms), Gα_12_, Gα_13_, and Gα_q_ were expressed by *GBM #4* and *GBM #10* at different levels. It is well known that GPR55 can be coupled to the Gα_12/13_ and Gα_q_ proteins. Both subunits were found in *GBM #4* and *GBM #10* at different levels of transcription (Figure 4a,b, Figures S5 and S6). Remarkably, Gα_q_ was highly expressed by *GBM #10* when compared to *GBM #4*, leading to an altered expression abundance of *GNAQ* compared to other transcripts of Gα subunits (Figure 4b and Figure S7). As PLC-dependent and RhoA/ROCK-independent signaling were observed in *GBM #4* (Figure 1a,b, Figure 2a,b, Figures S3a–c and S4a–c, Tables S2–S4), Gα_q_-mediated signaling by GPR55 was assumed. In contrast, in *GBM #10*, both RhoA/ROCK- and PLC-dependent signaling (Figure 1a,b, Figure 2a,b, Figures S3a–c and S4a–c, Tables S2–S4) were suggested via Gα_12/13_ and/or Gα_q_ proteins.

**Figure 5:**
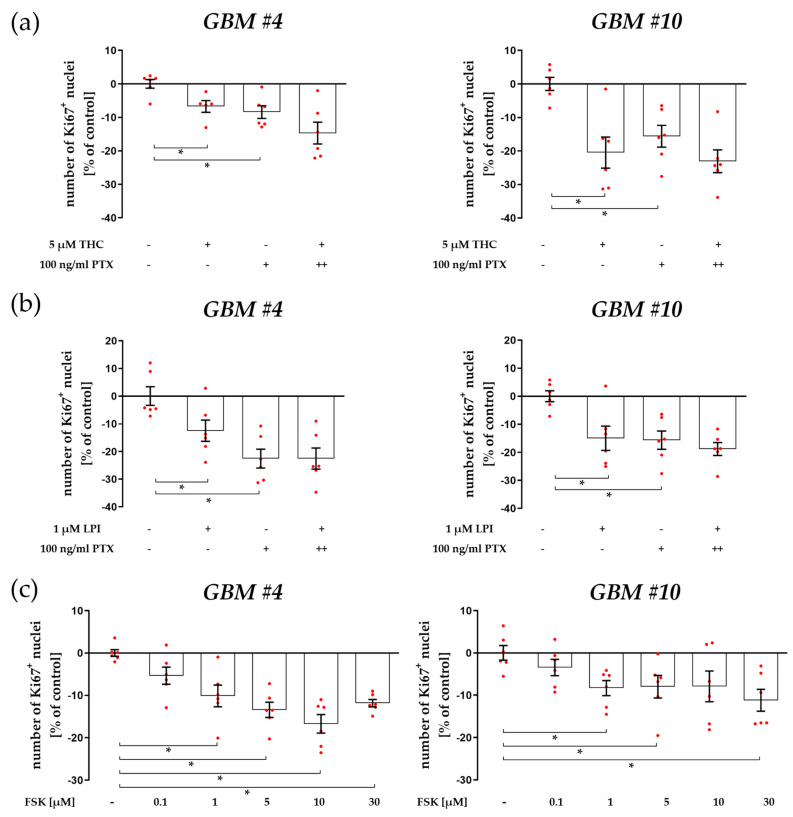
Impacts of pertussis toxin (PTX, Gα_o/i_ inhibitor) and forskolin (FSK) on the number of Ki67^+^ cells in the presence or absence of THC or LPI. *GBM #4* and *GBM #10* were left untreated or exposed to THC (**a**) or LPI (**b**) for 24 h, resulting in a decreased number of Ki67^+^ nuclei. A significantly decreased number of Ki67^+^ nuclei was detected after stimulation with PTX alone in *GBM #4* and *GBM #10*. When THC (**a**) or LPI (**b**) were applied after PTX pre-incubation, neither inhibitory nor additive effects were observed. In *GBM #4* and *GBM #10*, the number of Ki67^+^ nuclei was reduced concentration dependently after FSK stimulation for 24 h (**c**). FSK was applied in an ascending concentration series of 0.1 µM, 1 µM, 5 µM, 10 µM, and 30 µM. Significant effects were measured after incubation with ≥1 µM FSK. Data are means ± SEMs of N = 3 independent experiments performed in duplicate. Each red dot represents an individual data point. −/+ indicates without/with the corresponding substance. ++ denotes that cells were pre-incubated with PTX before THC or LPI was added. Significance was set at *p* < 0.05. The asterisk denotes significant results regarding the respective measurement indicated by the bar.

According to heterodimerization with other GPCRs, including CB_1_ and CB_2_, GPR55 might also activate other intracellular G-proteins such as Gα_i/o_ indirectly. Notably, *GBM #4* showed a significantly higher amount of Gα_o_ transcripts than *GBM #10*, leading to an altered expression abundance of *GNAOI* compared to other G-protein transcripts (Figure 4b). The involvement of Gα_i/o_ proteins as reported for CB_1_/CB_2_-dependent signaling was examined using the pertussis toxin (PTX), which inhibits the coupling of Gα_i/o_ proteins to their cognate GPCRs. PTX was unable to block actions of THC and LPI in *GBM #4* and *GBM #10*. Notably, PTX (100 ng/mL) displayed by itself an altered number of Ki67^+^ nuclei (Figure 5a,b, Table S8). Furthermore, no additive agonistic or antagonistic effects of PTX and THC or LPI were observed (Figure 5a,b, Table S8). Since blocking the Gα_i/o_ proteins with PTX leads to unhindered activation of adenylyl cyclase (AC), the increased AC activity was assumed to be responsible for the reduced number of Ki67^+^ nuclei after PTX treatment. To clarify the role of activated AC, increasing concentrations of forskolin (FSK) were applied (Figure 5c, Table S9). FSK reduced the number of Ki67^+^ nuclei in a concentration-dependent manner in *GBM #4* and *GBM #10*. The data confirm the participation of an increased AC activity in reducing the number of Ki67^+^ nuclei after PTX treatment that is independent of GPR55.

**Figure 6:**
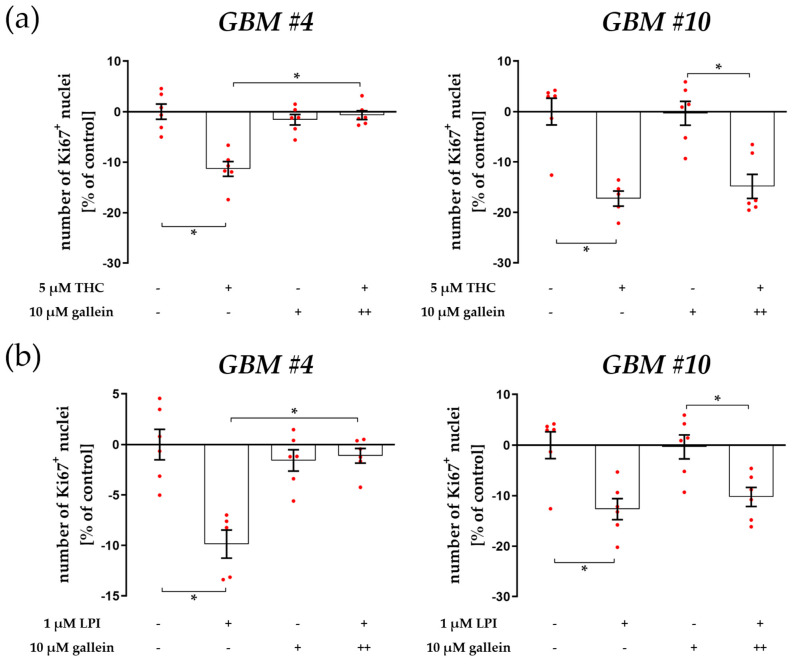
Impact of Gβγ inhibitor gallein on THC- and LPI-induced reduction of the number of Ki67^+^ nuclei. *GBM #4* and *GBM #10* were left untreated or exposed to THC (**a**) or LPI (**b**) for 24 h, resulting in a decreased number of Ki67^+^ nuclei. After cells were pre-incubated with gallein, responses to THC (**a**) and LPI (**b**) were significantly abolished in *GBM #4*. In contrast, in *GBM #10*, gallein caused no impact on THC- (**a**) and LPI-mediated signaling (**b**), reducing the number of Ki67^+^ nuclei. No alterations were observed when cells were stimulated with gallein alone (**a**,**b**). Data are means ± SEMs of N = 3 independent experiments performed in duplicate. Each red dot represents an individual data point. −/+ indicates without/with the corresponding substance. ++ denotes that cells were pre-incubated with gallein before THC or LPI was added. Significance was set at *p* < 0.05. The asterisk denotes significant results regarding the respective measurement indicated by the bar.

Since GPCRs have the capacity to produce signals through the actions of liberated Gα and Gβγ subunits, we further examined the involvement of Gβγ subunits. Targeting Gβγ subunits by gallein, a small molecule that binds to Gβγ and disrupts Gβγ signaling, significantly reversed the decreased number of Ki67^+^ nuclei elicited by THC in *GBM #4* (Figure 6a and Figure S3d, Table S10). In contrast, THC’s effect on *GBM #10* remained unaffected in the presence of gallein (Figure 6a and Figure S2d, Table S10). Similar results were obtained for LPI, as gallein attenuated the response to LPI in *GBM #4*, but not in *GBM #10* (Figure 6b, Figure S4d, Table S10). The data suggest that Gβγ-dependent (*GBM #4*) and Gβγ-independent (*GBM #10*) signaling might follow an activation of GPR55 by THC or LPI. 

### 3.3. Cell-Type-Specific Calcineurin Signaling and an Unaltered Subcellular Localization of NFAT 

**Figure 7:**
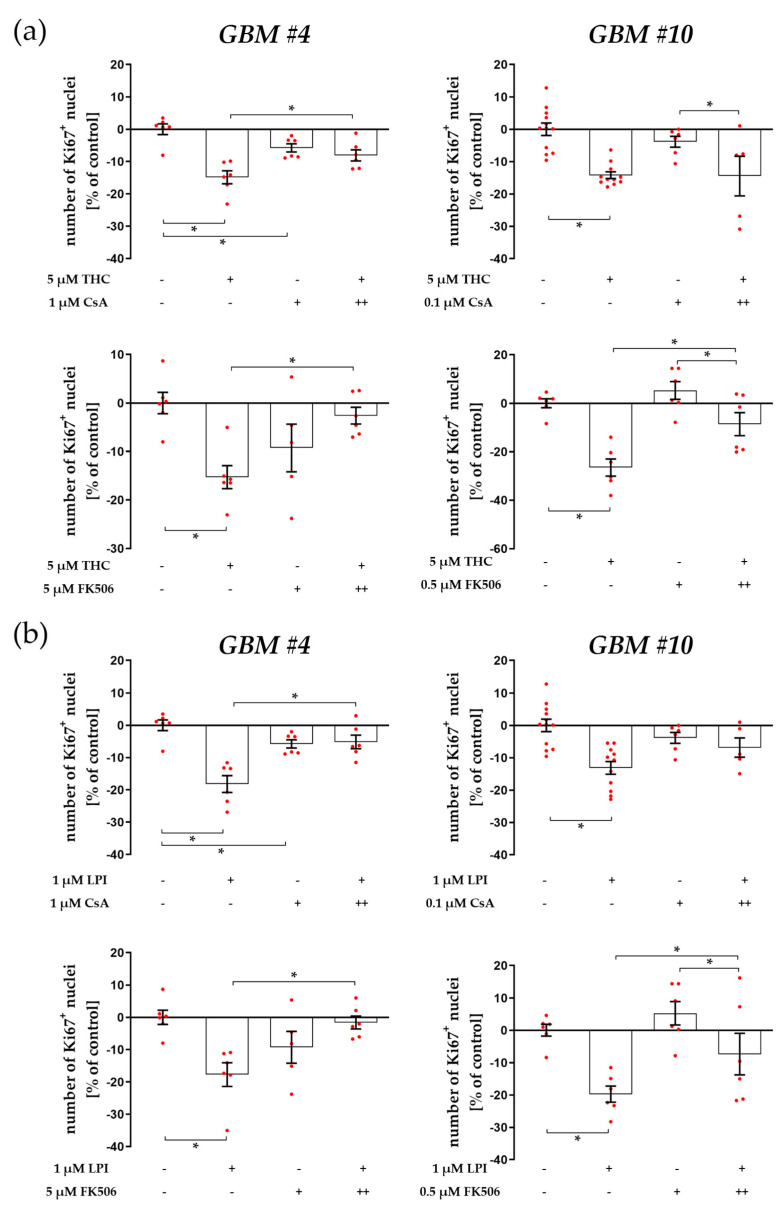
Impact of calcineurin inhibitor Cyclosporine A (CsA) and FK506 on THC- and LPI-induced reduction in the number of Ki67^+^ nuclei. *GBM #4* and *GBM #10* were left untreated or exposed to THC (**a**) or LPI (**b**) for 24 h, resulting in a decreased number of Ki67^+^ nuclei. In *GBM #4,* the effects of THC (**a**) and LPI (**b**) were significantly reduced by CsA and FK506, but CsA alone elicited a decreased number of Ki67^+^ nuclei compared to the untreated control group. In *GBM #10*, lower concentrations of CsA and FK506 were used. No significant effects on responses to THC (**a**) and LPI (**b**) were observed in the presence of CsA, whereas FK506 partially inhibited the effects of THC (**a**) and LPI (**b**). Data are means ± SEMs of N = 3 or N = 5 (*GBM #10*, CsA) independent experiments performed in duplicate. Each red dot represents an individual data point. -/+ indicates without/with the corresponding substance. ++ denotes that cells were pre-incubated with CsA or FK506 before THC or LPI was added. Significance was set at *p* < 0.05. The asterisk denotes significant results regarding the respective measurement indicated by the bar.

Well-established downstream effects of GPR55-driven PLC-IP3 signaling include an increase in intracellular Ca^2+^ concentrations and the Ca^2+^-dependent activation of calcineurin. Cyclosporine A (CsA) and FK506 were therefore used to bind to calcineurin and inhibit its phosphatase activity by forming complexes with immunophilins. In *GBM #4*, CsA and FK506 significantly attenuated the reduced number of Ki67^+^ nuclei after THC or LPI treatment (Figure 7a,b, Figures S3e,f and S4e,f, Tables S11 and S12). Notably, CsA treatment itself led to a decreased number of Ki67^+^ nuclei but to a smaller extent than observed for THC or LPI (Figure 7a,b and Figure S3e,f, Table S11). In contrast, in *GBM #10*, the effects of THC and LPI were not affected by CsA but were inhibited by FK506. It should be noted that in *GBM #10*, lower concentrations of CsA and FK506 had to be used as compared to *GBM #4*. 

**Figure 8:**
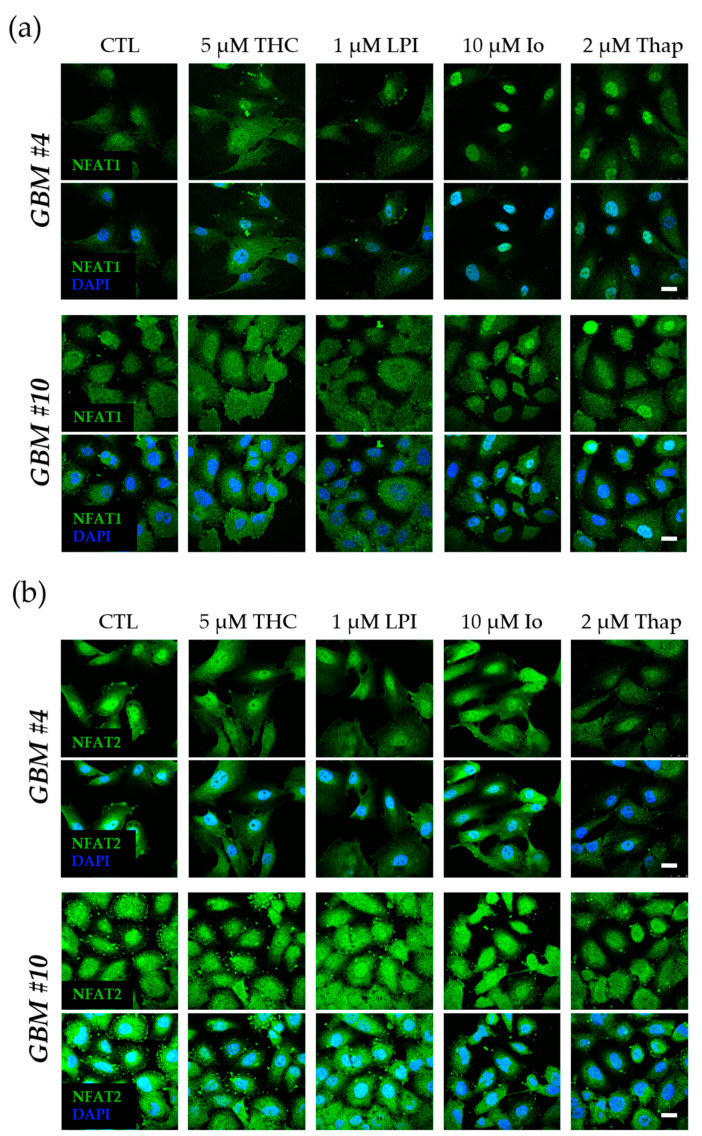
Influence of THC and LPI on the subcellular localization of NFAT1 and NFAT2 after 30 min. Representative images of NFAT1 (**a**) and NFAT2 (**b**) after 30 min of THC and LPI stimulation. In untreated control cells, NFAT1 (**a**) and NFAT2 (**b**) were localized in both the cytoplasm and nucleus. Translocation of NFAT1 (**a**) and NFAT2 (**b**) after THC or LPI administration was not detectable in *GBM #4* or *GBM #10*. Increased signals of nuclear NFAT1 (**a**) were observed after ionomycin (Io) and thapsigargin (Thap) in both cell lines. In contrast, signals of NFAT2 (**b**) remained unchanged. Cell nuclei were counterstained with DAPI. Scale bar = 25 µm.

**Figure 9:**
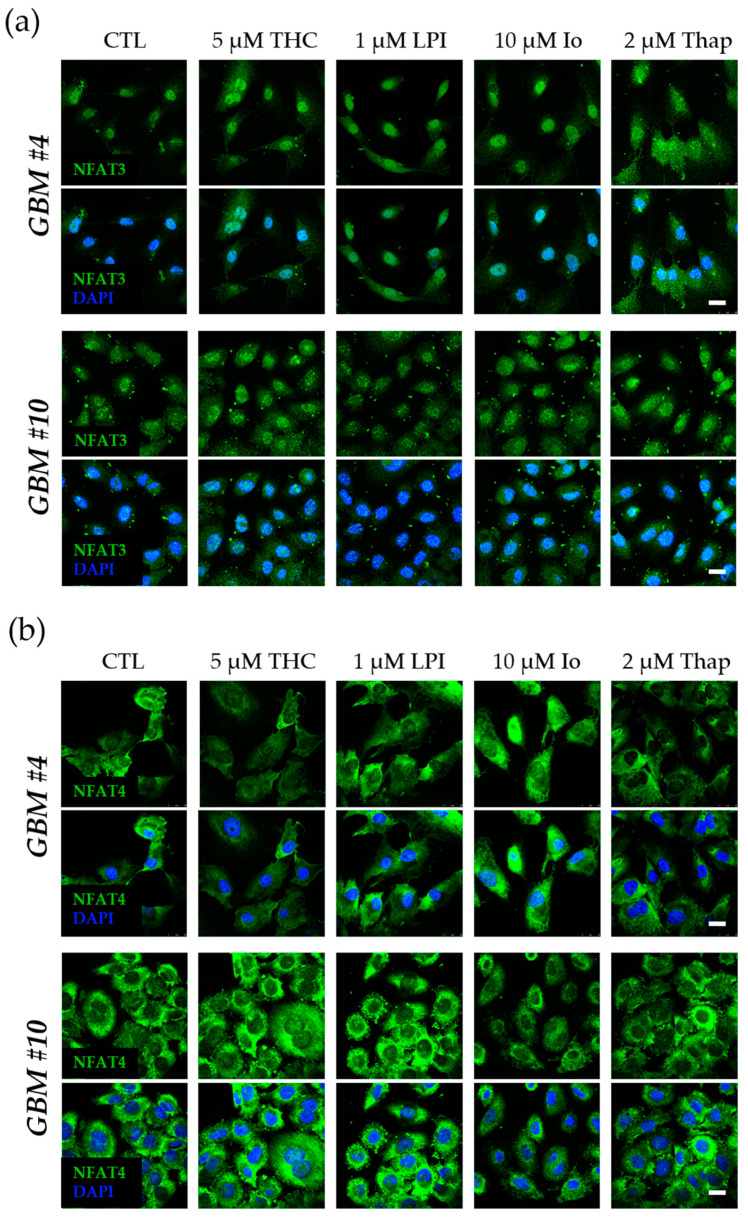
Influence of THC or LPI on the subcellular localization of NFAT3 and NFAT4 after 30 min. Representative images of NFAT3 (**a**) and NFAT4 (**b**) after 30 min of THC or LPI stimulation. In untreated control cells, NFAT3 (**a**) was mainly localized in the nucleus, and NFAT4 (**b**) was solely localized in the cytoplasm. Translocation of NFAT3 (**a**) and NFAT4 (**b**) after THC, LPI, ionomycin (Io), and thapsigargin (Thap) administration was not detectable in *GBM #4* and *GBM #10*. Cell nuclei were counterstained with DAPI. Scale bar = 25 µm.

Next, we tested the ability of GPR55 activation by THC and LPI to induce NFAT as an example of a Ca^2+^/calcineurin-dependent regulator of transcription. Given that NFAT5 is generally associated with responses to osmotic stresses independently of calcineurin, its role in response to THC and LPI was not examined. Calcineurin-dependent dephosphorylation of NFAT promotes the translocation of cytoplasmic NFAT into the nucleus to initiate or repress transcription of NFAT-specific genes. Resident inactive NFAT is localized within the cytoplasm, whereas dephosphorylated, activated NFAT is found in the nucleus. However, NFAT1-4 was detected in the nucleus and cytoplasm of *GBM #4* and *GBM #10* (Figure 8 and Figure 9). Subcellular localization of different NFAT isoforms was visualized by fluorophore labeling (Figure 8 and Figure 9). In untreated control cells, NFAT1, NFAT2, and NFAT4 were mainly detected in the cytoplasm (Figure 8 and Figure 9). NFAT1 and NFAT2 at low basal levels and NFAT3 at high levels were also localized in the nucleus. In contrast, NFAT4 was observed solely in the cytoplasm (Figure 9b). Stimulation with THC or LPI did not produce significant changes in the nuclear signals of NFAT1, NFAT2, NFAT3, or NFAT4 in *GBM #4* and *GBM #10* after 5 min, 10 min, 30 min, 2 h, 4 h, or 24 h (NFAT1 in *GBM#4* Figure S8).

**Figure 10:**
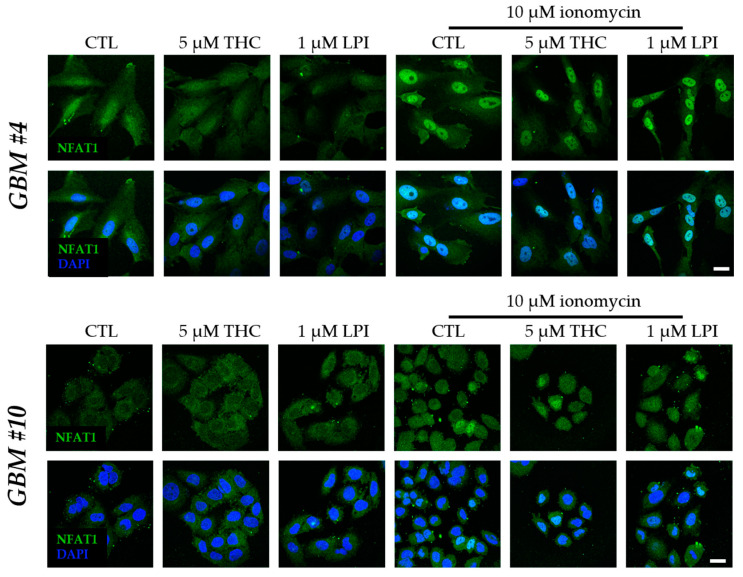
Influence of ionomycin on the subcellular localization of NFAT1 after 30 min in the presence of THC and LPI. THC and LPI had no effect on the subcellular localization of NFAT1 in *GBM #4* and *GBM #10*. Ionomycin (Io) induced a marked translocation of NFAT1 into the nucleus after 30 min. In the presence of THC or LPI, ionomycin’s effects remained unchanged. Cell nuclei were counterstained with DAPI. Scale bar = 25 µm.

In contrast, ionomycin (Io, 10 µM) and thapsigargin (Thap, 2 µM), which both increased the intracellular calcium level via different mechanisms, led to an altered localization of NFAT1 after 30 min in *GBM #4* and *GBM #10*, reflected by higher nuclear signals of NFAT1 (Figure 8a and Figure 10). Interestingly, localization of NFAT2, NFAT3, and NFAT4 remained unaffected by Io and Thap (Figure 8b and Figure 9). 

In addition, THC or LPI did not affect Io-induced translocation of NFAT1 into the nucleus (Figure 10). However, Io effects were fully blocked by calcineurin-inhibitors FK506 and CsA (Figure S9), indicating a calcineurin-dependent translocation of NFAT1 into the nucleus after Io treatment. Furthermore, the data confirm that the concentrations of FK506 and CsA used produced sufficient inhibition of calcineurin. 

Remarkably, single stimulation with FK506 and CsA did not produce changes in nuclear signals of constitutively active NFAT2 and NFAT3 (Figure S10), suggesting calcineurin-independent activation of NFAT2 and NFAT3 or a low rate of NFAT nuclear exportation. Altogether, the data indicate an NFAT-independent regulation of the number of Ki67^+^ nuclei after THC or LPI treatment.

## 4. Discussion

The endocannabinoid system in general and cannabinoids in particular, like plant-derived THC and CBD, are believed to be promising targets and compounds for additional treatment strategies against highly malignant glioblastomas. They exert tumor-suppressive properties by inhibiting proliferation or inducing apoptosis, which are mostly mediated by cannabinoid-specific receptors such as CB_1_ or CB_2_ [14,18,19,20]. Based on their complex pharmacology, cannabinoids may also modulate cellular responses independently of classical CB_1_/CB_2_ receptors via other receptors such as GPR55 [2,22]. We recently discovered that THC affected the number of Ki67^+^ nuclei from patient-derived cells of human glioblastomas by GPR55 [16]. The focus of the present study was to characterize a part of the signaling pathway downstream of GPR55 that contributes an altered number of Ki67^+^ nuclei after THC or LPI treatment. 

### 4.1. The Involvement of PLC-IP3 and RhoA-ROCK Signaling Pathways

GPR55 is thought to bind to Gα_12/13_ or Gα_q_ rather than Gα_s_ or Gα_i/o_ proteins [1,2,25]. Stimulation of Gα_q_ proteins is generally followed by an activation of PLC [2]. Studies on cell lines and primary cells of different origins revealed that pharmacological activation of GPR55 by LPI and other cannabinoids led to increased intracellular Ca^2+^ concentrations ([Ca^2+^]_i_) via the activation of PLC and formation of IP3 [2,25,36,38]. In HEK293 cells and neurons of dorsal root ganglia, a PLC-IP3-dependent increase in [Ca^2+^]_i_ was observed after stimulation with 5 µM THC and 3 µM LPI [2]. The data of the present work indicate that the THC- and LPI-mediated reduction of Ki67^+^ nuclei might follow a PLC- and IP3-dependent mechanism via GPR55. PLC-IP3-dependent signal transductions through the activation of CB_1_ and CB_2_ receptors have so far not been reported for THC. In CB_1_-transfected HEK293 cells, stimulation with 10 µM THC had no measurable effects on [Ca^2+^]_i_, although CB_1_-dependent Ca^2+^ release via WIN 55,212-2 was detected [39]. These results are highly consistent with CB_1_/CB_2_-independent effects of THC despite the presence of CB_1_ receptors as reported previously [16] and support GPR55-specific signaling via PLC and IP3. 

In addition, GPR55-mediated RhoA activation by LPI and cannabinoids was reported in HEK293 and PC12 cells [1,2,25,26,36]. RhoA-ROCK was shown to be activated downstream of Gα_12/13_ and to a lesser extent Gα_q_. Since many cellular processes, such as proliferation or migration, might be regulated by RhoA and ROCK, the involvement of RhoA via the inhibition of ROCK was here investigated. A cell-type-specific RhoA-ROCK signaling was observed, indicating differences in intracellular coupling of G proteins to GPR55. Interestingly, in *GBM #10*, the reduction of the Ki67^+^ nuclei after THC and LPI stimulation might be explained by the interplay between PLC and RhoA-ROCK signaling pathways. In endothelial cells of a rat mesenteric arterial bed, an interaction between PLC- and RhoA-ROCK-modulated [Ca^2+^]_i_ was found after GPR55 activation by LPI or AM251 [36]. The release of intracellular Ca^2+^ was characterized by two phases. Inhibition of PLC by U73122 blocked the initial phase of Ca^2+^ release, whereas the late phase was abolished by inhibiting ROCK via Y-27632, suggesting a biphasic mechanism [36]. Both phases were abolished by blocking IP3-sensitive receptors with 2-APB, indicating ROCK-induced IP3 generation by direct PLC activation [36]. These data suggest a similar mechanism in *GBM #10* after THC or LPI stimulation, involving both PLC- and ROCK-dependent signaling. 

The precise mechanism of ROCK-regulated PLC is not fully understood. In GPR55-expressing HEK293 cells, the actin cytoskeleton has been shown to play a pivotal role in the generation of GPR55-dependent Ca^2+^ signals. The family of Rho proteins, including RhoA, regulates protein biosynthesis and cell proliferation as well as the organization of the actin cytoskeleton by affecting actin stabilization through activation of LIM kinase or actomyosin contraction via MLC (modulator of VRAC current 1) [40]. RhoA activation and an intact actin cytoskeleton are required for the THC (5 µM) mediated rise in Ca^2+^ in HEK293 cells [2]. In contrast, pure PLC-mediated pathways as found upon the activation of muscarinic acetylcholine receptors of the parasympathetic nervous system were not affected by either RhoA inhibition or deteriorated actin cytoskeleton formation [40]. Therefore, an intact actin cytoskeleton might play a central role in modulating [Ca^2+^]_i_ when GPR55 is coupled to RhoA-ROCK signaling. In murine fibroblast cell line NIH 3T3, a disrupted cytoskeleton altered the spatial interaction between PLC and IP3-sensitive receptors, thus affecting PLC-dependent Ca^2+^ signaling [41]. Taken together, these data suggest that the RhoA-ROCK-signaling pathway may indirectly promote PLC-dependent reduction of Ki67^+^ nuclei in *GBM #10* through alterations in the spatial location of PLC- and IP3-sensitive receptors. The requirement for direct ROCK activation is supported by the ROCK-independent signaling in *GBM #4*, making the involvement of basal-active ROCK not evident. 

### 4.2. Cell-Type-Dependent Coupling of GPR55 to Gα_12/13_ and/or Gα_q_ and the Role of Gβγ

The divergence in RhoA-ROCK involvement suggests cell-type-specific differences in coupling to intracellular G proteins. Generally, RhoA-ROCK signaling is known to be activated downstream of Gα_12/13_, whereas PLC activation is responsive to Gα_q_ pathways [2]. Previous studies implicated intracellular coupling of GPR55 to Gα1_2/13_ and/or Gα_q_ pathways [1,2,30]. Missing effects of the ROCK inhibitor in *GBM #4* indicated a coupling of GPR55 signaling to Gα_q_. In contrast, in *GBM #10*, GPR55-dependent signaling might be related to Gα_12/13_ coupling or dual signaling via Gα_q_ and Gα_12/13_ due to PLC- and ROCK-dependent signaling. Comparable observations were conducted in GPR55-expressing HEK293 cells [25], in neurons of the dorsal root ganglia [2], and in mesenteric arterial endothelial cells [36], where a PLC- and RhoA-ROCK-dependent increase in [Ca^2+^]_i_ was measured. Moreover, increased [Ca^2+^]_i_ in the neurons of dorsal root ganglia after GPR55 activation was attributed to dual Gα_12/13_ and Gα_q_ signaling [2]. Some GPCRs functionally switch their associated subfamily of G proteins depending on the expression level of G proteins [39,42]. In the present work, differences in the expression of the relevant Gα_q_ and Gα_12_ subunits were found but did not explain cell-type-specific signaling. 

GPCRs can initiate signaling cascades not only via Gα subunits. Dissociation of the trimeric complexes after receptor activation also releases free and active Gβγ subunits that might be relevant for signal transduction events. Thus, the aspect of Gβγ-dependent signaling was further examined. A cell-type-specific Gβγ dependence was found. A correlation between Gβγ signaling and GPR55 has so far not been demonstrated in tumor cells. In murine slice cultures of the substantia nigra, GPR55 signaling induced by LPI or O-1602 was associated with the presynaptic release of [^3^H] gamma-amino-butyric acid (GABA) by a Gβγ-dependent mechanism [43]. In contrast to the present study, Gβγ-mediated AC activation with a subsequent cAMP accumulation was assumed to be an underlying mechanism [43], reflecting species- and cell-type-specific differences.

Although all isoforms of G proteins release one molecule of a Gβγ dimer per activation event, Gβγ signaling is strongly linked to Gα_i/o_-derived Gβγ subunits [44]. It is known that GPR55 does not interact with Gα_o/i_ proteins. Alternatively, GPR55 is able to form heterodimers with Gα_o/i_-coupled receptors, as described for CB_1_ [45], CB_2_ [46,47], and LPA2 [48], resulting in an altered activity and signal transduction of GPR55. To test the involvement of Gα_o/i_ proteins, PTX was used, as it prevents signal transduction of Gα_o/I_ by catalyzing irreversible ADP-ribosylation of the α subunit of trimeric Gα_o/i_βγ complexes [49]. In our study, PTX was found to be unsuitable for analyzing Gα_o/i_-dependent signaling because it directly affected the number of Ki67^+^ nuclei. The responses to PTX could be explained by an accumulation of cAMP due to an enhanced cAMP production via stimulatory GPCRs and an unhindered activation of cAMP-mediated signaling pathways. As an alternative mechanism, Gα_o/i_-independent effects of PTX [50] should be considered, which occurred after using high concentrations of PTX. Direct stimulation of AC and cAMP production by FSK reduced the number of Ki67^+^ nuclei comparable to PTX, demonstrating unhindered activation of cAMP-mediated signaling pathways and confuting the Gα_o/i_-independent effects of PTX. As PTX treatment showed high background activity, the role of Gα_o/i_-derived Gβγ in GPR55 signaling in *GBM #4* remains an open question.

Gβγ subunits are able to stimulate PLC directly or indirectly via PI3K [29,51,52,53,54]. Notably, Gα_q_-derived Gβγ subunits also have the capacity for signaling but to a lesser extent than those derived from Gα_i/o_ and might generate signals via PI3K activation [55]. PI3K and PI3K-dependent PLC activation were described as mechanisms for GPR55-driven Ca^2+^ signaling in endothelial cells after AEA treatment [29]. Thus, in accordance with Gα_q_ signaling in *GBM #4*, Gα_q_-derived Gβγ signaling via PI3K could be conceivable. Therefore, the involvement of PI3K should be investigated in further studies. 

Alternatively, it was reported that Gβγ subunits might influence the extent of Gα_q_-dependent signaling through direct interaction with PLC [51,53,54]. On the one hand, Gβγ subunits were able to directly stimulate the activity of PLC [51,53] and thus act synergistically with the Gα_q_-dependent stimulation of PLC [52,53,54]. On the other hand, Gβγ subunits inhibit the Gα-GTPase activity of PLC, as PLC is both an effector of Gα_q_ and a Gα_q_-selective GTPase-activating protein (GAP) [53,56]. Since prevention of Gβγ disrupts interaction with their downstream effectors, it is conceivable that stimulation of PLC by Gα_q_ alone was insufficient to exert effects after THC or LPI application. Therefore, in the case of *GBM #4*, a reduced number of Ki67^+^ nuclei by GPR55 might be determined by the duration and strength of PLC stimulation through interplay between Gα_q_ and functional Gβγ subunits. The divergence of signaling observed in *GBM #10* might base on the coupling of GPR55, as *GBM #10* Gα_12/13_ and ROCK activation directly or indirectly contributed to a sufficient duration and strength of PLC activation and/or downstream signals to modulate the number of Ki67^+^ nuclei. As discussed previously, this is in line with a biphasic increase in [Ca^2+^]_i_ by PLC and ROCK signaling in rat mesenteric arterial bed endothelial cells stimulated with LPI or AM251 [36]. It remains to be determined whether the functionality of GPR55, its Gβγ, and RhoA-ROCK-dependent signaling and responsiveness to THC or LPI might be used as a positive prognostic marker.

### 4.3. Cell-Type-Specific Calcineurin Signaling and the Role of NFAT

Due to the involvement of IP3-sensitive receptors, a Ca^2+^-dependent process was assumed in the present study [57]. Ca^2+^ functions as second messenger and activates various Ca^2+^-dependent protein kinases and calcineurin, a Ca^2+^/calmodulin-dependent protein phosphatase [58]. These enzymes act as transducers for transmitting Ca^2+^ signals from the cytosol to the nucleus. While Ca^2+^-dependent protein kinases activate transcription factors, including CREB or NF-κB, by phosphorylation, calcineurin catalyzes dephosphorylation of the resident cytoplasmic transcription factor, such as NFAT [59]. Subsequently, NFAT translocates into the nucleus and induces or represses NFAT-specific genes. It is known that GPR55 is linked to an increased activity of NFAT via elevation of [Ca^2+^]_I_ [25,30]. To investigate the possibility of a calcineurin-dependent mechanism after THC and LPI treatment, we used two non-competitive calcineurin inhibitors (CsA and FK506), which limited the access of peptide and protein substrates to the active site of calcineurin [60,61]. Calcineurin-dependent signaling was observed in a cell-type-specific manner. Interestingly, partial inhibition of THC- and LPI-mediated effects in *GBM #10* after FK506 treatment was evident, whereas CsA had no detectable influence. This phenomenon might be explained by a higher binding affinity of FK506 complexes to calcineurin compared to CsA complexes [62]. 

However, no alterations in the subcellular localization of different isoforms of NFAT, namely NFAT1-4, were detected after THC or LPI stimulation. This is in strong contrast to calcineurin-dependent signaling in *GBM #4* but supports partial calcineurin-independent effects observed in *GBM #10*. The pronounced background activity of CsA and FK506 did not enable a clear conclusion and was unlikely to be attributable to the inhibition of basally active NFAT. After CsA and FK506 treatment, NFAT2 and NFAT3 were constitutively present in the nucleus, suggesting calcineurin-independent mechanisms in these tumor cells. Notably, the effects of both are not limited to calcineurin but also regulate the transcriptional activity of NF-κB or proteasomal degradation [63].

The induction of NFAT in general and NFAT4 in particular was reported after LPI-driven GPR55 activation [25,29,64]. The differences compared to our study might be explained by the experimental conditions used, such as an artificial overexpression of GPR55 in HEK293 or serum starvation before and during stimulation experiments. Both conditions facilitate efficient NFAT activation by a prolonged Ca^2+^ stimulus due to increased responses resulting from the overstimulation of ectopic GPR55 expression and the absence of inhibitory components on Ca^2+^ signals that are present in the medium-containing serum. These conditions seemed to be absent in the glioblastoma cells investigated in this study. The intensity and duration of Ca^2+^ signals generated by store-operated calcium channels, such as IP3-sensitive receptors, play a crucial role in NFAT activation [59]. The activation of NFAT1 and NFAT4 required different strong and subcellular Ca^2+^ signals [65]. NFAT1 was selectively recruited at low stimulus intensities, whereas activation of both isoforms occurs with increasing receptor occupancy and continuous Ca^2+^ influx. Furthermore, different kinetics of the nuclear export of NFAT1 and NFAT4 were revealed. The slow export of NFAT1 allows activation of gene expression even in the presence of low-frequency Ca^2+^ spikes because NFAT1 remains in the nucleus longer after Ca^2+^ signals are terminated. In contrast, NFAT4 was only effective when Ca^2+^ mobilization was sustained, as its export was very rapid [65]. In the present study, we observed higher nuclear signals of NFAT1 after ionomycin or thapsigargin treatment, which both generated acute Ca^2+^ signaling [66,67]. In contrast, signals of NFAT2-4 remained unaffected. To obtain nuclear signals of NFAT2-4, co-treatment for inducing cooperation partners of NFAT such as c-Fos and c-Jun is likely needed. In NFAT overexpressing HEK293 cells, NFAT4-dependent luciferase expression was increased in response to ionomycin and PKC-activating PDBu but not to ionomycin alone [68]. Furthermore, NFAT4 showed pulsatile translocation dynamics, as found in mast cells [69].

However, in comparison to acute Ca^2+^ signals by ionomycin or thapsigargin, THC and LPI were unable to induce NFAT1 translocation. The requirement of a more prolonged Ca^2+^ stimulus to overcome glycogen synthase kinase 3 beta (GSK3β) activity, which persistently phosphorylates NFAT and counteracts calcineurin dephosphorylating activity, might be a possible scenario for efficient NFAT1 activation.

Altogether, the data suggest that NFAT did not participate in the reduction of Ki67 after THC or LPI treatment. Furthermore, cell-type-specific calcineurin signaling gives rise to speculation that other calcineurin-dependent and calcineurin-independent signaling pathways are involved and react more sensitively to transient Ca^2+^ signals. Alternatively, basal NFAT activation, which was abrogated by FK506 and CsA, might be needed when it is acting as a coactivator for another transcriptions factor, which becomes active after THC and LPI treatment. However, in addition to NFAT, calcineurin also dephosphorylates other transcription factors, including CREB-regulated transcriptional coactivator (CRTC) 1 [70], transcription factor EB (TFEB) [71], myocyte enhancer factor 2 (MEF2) [72], and ETS Like-1 (ELK-1) [58]. Both CsA and FK506 might inhibit the transcriptional activity of NF-κB, which may in turn became activated by Ca^2+^/calmodulin-dependent protein kinase II (CaMKII). The mechanism behind the role of NF-κB should further be explored as a calcineurin-independent mechanism.

Similar cell-type-dependent effects were observed in cAMP-mediated signaling pathways induced by PTX and FSK, which give rise to speculation of additional signaling pathways capable of reducing the number of Ki67^+^ nuclei. Here, the possible involvement of CREB should be highlighted. Although CREB is not a direct substrate for calcineurin, it might indirectly be regulated by calcineurin or by Ca^2+^ signals. For instance, calcineurin is a part of a negative feedback loop of Ca^2+^-initiated transcription of CREB genes [73]. Interestingly, an increase in [Ca^2+^]_i_ activates CREB-directed gene transcription via activation of CaMKIV and subsequently induces inactivation of CREB via calcineurin-mediated activation of protein phosphatase-1 [73]. Alternatively, calcineurin can operate as a positive regulator of CREB by promoting nuclear translocation of CREB-regulated transcriptional co-activators (CRTCs) [70]. Under basal conditions, CRTCs retain their phosphorylated form in the cytoplasm by interacting with 14-3-3 proteins. Both cAMP and Ca^2+^ signals can induce dephosphorylation of CRTCs by inhibition of salt-inducible kinases (SIKs, which phosphorylate CRTCs) or by the induction of calcineurin, respectively. Dephosphorylated CRTC translocates into the nucleus, binds to the bZIP domain of CREB, and operates as a co-activator [70]. The responsible underlying mechanism for an altered Ca^2+^-dependent CREB activation is still not understood but might result from cell-type-specific differences and the amplitude, duration, and subcellular localization of Ca^2+^ signals [58]. These hypotheses should be considered in future investigations, including analyses of phosphorylation state, activity, and nuclear localization of CREB and CRTCs.

## 5. Conclusions

In the present work, signaling pathways related to GPR55 that might account for previously described THC-mediated reduction in the number of Ki67^+^ nuclei of patient-derived glioblastoma cells were investigated. A strong involvement of the PLC-IP3 pathway was observed. Cell-type differences in Gβγ and RhoA-ROCK signaling were found, probably explained by differences in the coupling of GPR55 to intracellular G proteins. Additionally, GPR55-mediated effects required at least partial activation of calcineurin. Calcineurin-related activation of transcription factor NFAT was not evident after THC or LPI treatment, as its subcellular localization remained unchanged. However, the analysis of additional transcription factors, which are influenced by IP3-driven Ca^2+^ release and/or calcineurin, should be elucidated in future investigations to shed light on the precise mechanism of the GPR55-mediated reduction of Ki67^+^ nuclei in glioblastoma cells. As the present study showed similarities and differences in GPR55-associated signaling compared to the literature, we may anticipate a better understanding of the complex pathways by which THC and GPR55 affect tumor cell biology in the context of glioblastomas. The data underline the diversity of GPR55-associated signaling pathways that are distinct in various cell types and from other cannabinoid receptors, including CB_1_ and CB_2_.

Furthermore, this diversity might account in part for the individual responsiveness of tumor cells to GPR55 stimuli by cannabinoids. Nevertheless, the identification of the precise signaling pathway might be useful for the purpose of finding prognostic markers and defining the conditions in which THC and GPR55 may be beneficial or unemployable as novel therapeutic options.

## Figures and Tables

**Table 1:** Substances.

Substances	Targets	Behavior	Solvent	Concentration	Pre-Incubation Time	Company	Article Number
2-APB	IP3-sensitive receptors	inhibitor	DMSO	10 µM	30 min [28]	Tocris, Bristol, UK	1224
cyclosporine A (CsA)	calcineurin	inhibitor	DMSO	0.1 µM 1 µM	1 h [31]	Tocris, Bristol, UK	1101
Dronabinol (THC)	CB_1_ CB_2_ GPR18 GPR55	agonist [22]	DMSO	5 µM	-	THC pharm GmbH, Frankfurt am Main, Germany	THC-1016
FK506	calcineurin	inhibitor	DMSO	0.5 µM 5 µM	1.5 h [32]	Tocris, Bristol, UK	3631
forskolin	adenylyl cyclase	activator	DMSO	0.1 µM 1 µM 5 µM 10 µM 30 µM	-	Sigma, Darmstadt, Germany	
gallein	Gβγ-subunits	inhibitor	DMSO	10 µM	15 min [33]	Tocris, Bristol, UK	3090
ionomycin	-	calcium ionophore	DMSO	10 µM	-	Tocris, Bristol, UK	1704
lysophosphatidyl-inositol (LPI)	GPR55	agonist [34]	DMSO	1 µM	-	Sigma, Darmstadt, Germany	L7635
pertussis toxine (PTX)	G_i/o_-proteins	inhibitor	H_2_O	100 ng/ml	16 h [35]	Tocris, Bristol, UK	3097
U73122	PLC	inhibitor	DMSO	0.1 µM	15 min [36]	Cayman Chemicals, Ann Arbor, Michigan, USA	70740
U73343	-	inactive analogue of U73122	DMSO	0.1 µM	15 min	Tocris, Bristol, UK	4133
thapsigargin	SERCA	inhibitor	DMSO	2 µM	-	Tocris, Bristol, UK	1138
Y-27632	ROCK	inhibitor	H_2_O	10 µM	1 h [37]	Tocris, Bristol, UK	1254

**Table 2:** Primers.

Gene	Accession Number	Forward Primer (5′-> 3′)	Reverse Primer (5′-> 3′)	Size [bp]
*GAPDH*	NM_002046	TGCACCACCAACTGCTTAGC	GGCATGGACTGTGGTCATGAG	87
*GNA12*	NM_007353	GAGCTCTGCAGGTGTGGATT	GAAGATGGGAGAGCCGTCTG	226
*GNA13*	NM_006572	CGTCGAGAATTTCAACTGGGTG	CTTTGGTGGGTCTTCTGGCA	121
*GNAI1*	NM_002069	GCTGAAGATGAAGAAATGAACCGAA	GTCCCAGATGCATTTGCCTT	481
*GNAI2*	NM_002070	CAGGCAGCTATTTGCACTGTC	AGGTCGTTCAGGTAGTAGGC	168
*GNAI3*	NM_006496	AGTTTCCGTGGTGTGAGTGA	GATTCTCCAGCACCGAGTAGC	184
*GNAO1*	NM_020988	TGGTGATAAGGAGAGAAAGGCTG	TCGTTGAGCTGATACTCCCG	168
*GNAQ*	NM_002072	TGAGCACAATAAGGCTCATGC	ATCTTGTTGCGTAGGCAGGT	226
*GNASL*	NM_000516	GAGCAACAGCGATGGTGAGA	TGATCGCTCGGCACATAGTC	342
*GNASS*	NM_080426	GCAGAAGGACAAGCAGGTCTA	TTGGTTGCCTTCTCACTGTCTC	141
*POLR2A*	NM_000937	CTTGCCCCGTGCCATGCAGA	CTCGCACCCGGCCTTCCTTG	83
*TBP*	NM_003194	GAGCTGTGATGTGAAGTTTCC	TCTGGGTTTGATCATTCTGTAG	117

**Table 3:** Antibodies.

Antibodies	Species	Concentration (Application)	Company	Article Number
anti-Ki67	rabbit	1:200 (ICC)	DSC innovative Diagnostic-System, Hamburg, Germany	KI681C002
anti-NFAT1	rabbit	1.200 (IF)	Cell Signaling, Danvers, MA, USA	5861
anti-NFAT2	rabbit	1:200 (IF)	Invitrogen, Schwerte, Germany	PA5-90432
anti-NFAT3	rabbit	1:200 (IF)	Invitrogen, Schwerte, Germany	PA1-021
anti-NFAT4	rabbit	1:200 (IF)	Invitrogen, Schwerte, Germany	PA5-99546
anti-rabbit Alexa488 conjugated	goat	1:200 (IF)	Invitrogen, Schwerte, Germany	A11034
anti-rabbit IgG, biotin conjugated	goat	1:100 (ICC)	Sigma, Darmstadt, Germany	B7389

## Data Availability

Data are contained within the article and supplementary materials. The data of the current study has not been deposited in a public repository but is available from the lead author on request.

## Supplementary Materials

The following supporting information can be downloaded at: https://www.mdpi.com/article/10.3390/cells12222646/s1.
