# Supplementary material for: Elucidation of GPR55-Associated Signaling behind THC and LPI Reducing Effects on Ki67-Immunoreactive Nuclei in Patient-Derived Glioblastoma Cells
Source: Cells. 2023 Nov 17;12(22):2646. doi: 10.3390/cells12222646 (PMC10670585; doi:10.3390/cells12222646)
Supplement: Supplementary file 1 [file cells-12-02646-s001.zip › cells-2575477-supplementary.pdf]

Table S1. Data in patients and primary tumor samples of investigated glioblastoma cells [modified from [16].

			GBM #4	*GBM #10*
primary tumor sample		sex	female	male
		age	50	56
		tumor location	temporooccipital	temporal
		tumor origin	*de novo*	*de novo*
		MGMT[1] promotor	methylated	methylated
		IDH1[2] status (R132H)	wildtype	wildtype
patient-derived cells		MGMT promotor	methylated	methylated
		IDH1 status (R132H)	wildtype	wildtype
	stem cell marker	CD133^{+} cells	< 1%	< 1%
		SOX2 mRNA	detectable	detectable
		MSI1 mRNA	detectable	detectable
		NES mRNA	detectable	detectable
		CD44 mRNA	detectable	detectable

[1]O-6-methylguanine-DNA methyltransferase; [2] isocitrate dehydrogenase 1

(a)

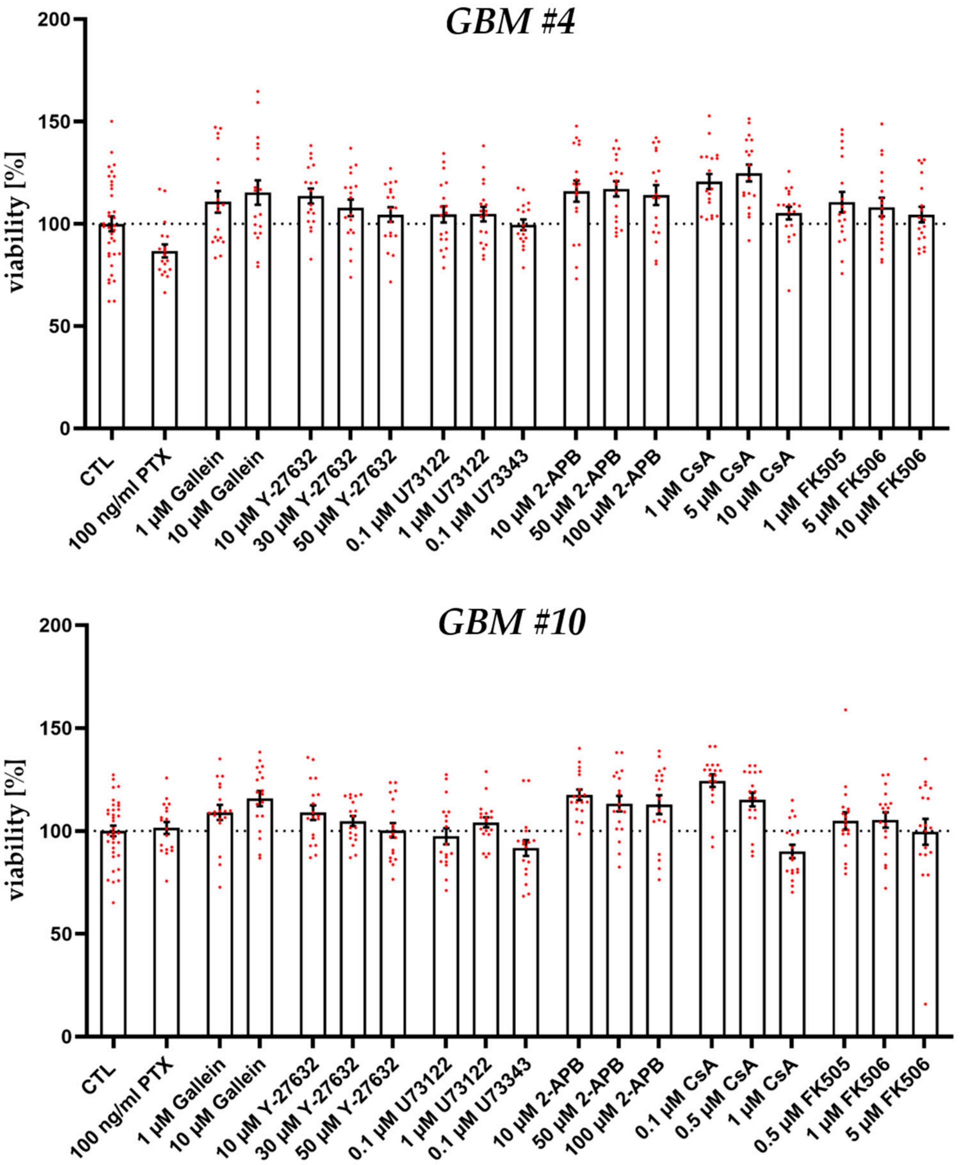
GBM #4
viability [%]
0
50
100
150
200
CTL
100 ng/ml PTX
1 µM Gallein
10 µM Gallein
10 µM Y-27632
30 µM Y-27632
50 µM Y-27632
0.1 µM U73122
1 µM U73122
0.1 µM U73343
10 µM 2-APB
50 µM 2-APB
100 µM 2-APB
1 µM CsA
5 µM CsA
10 µM CsA
1 µM FK505
5 µM FK506
10 µM FK506
GBM #10
viability [%]
0
50
100
150
200
CTL
100 ng/ml PTX
1 µM Gallein
10 µM Gallein
10 µM Y-27632
30 µM Y-27632
50 µM Y-27632
0.1 µM U73122
1 µM U73122
0.1 µM U73343
10 µM 2-APB
50 µM 2-APB
100 µM 2-APB
0.1 µM CsA
0.5 µM CsA
1 µM CsA
0.5 µM FK505
1 µM FK506
5 µM FK506

(b)

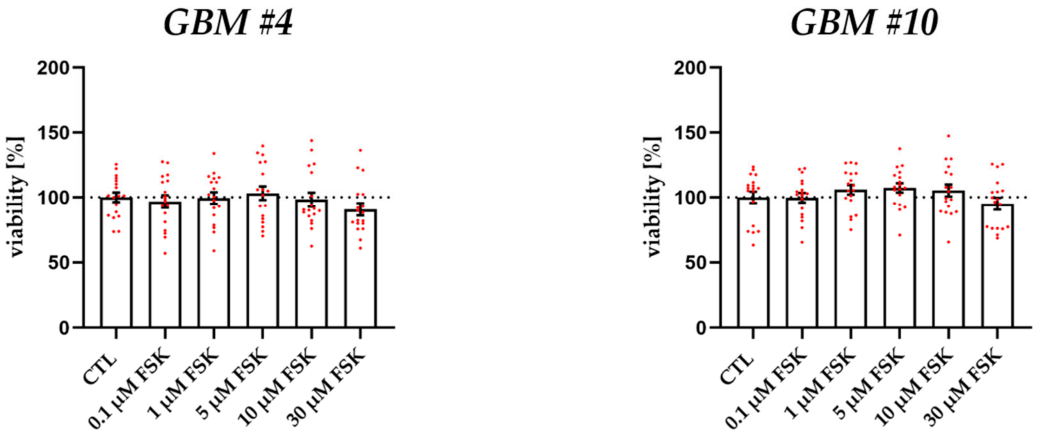
GBM #4
viability [%]
0
50
100
150
200
CTL
0.1 µM FSK
1 µM FSK
5 µM FSK
10 µM FSK
30 µM FSK
GBM #10
viability [%]
0
50
100
150
200
CTL
0.1 µM FSK
1 µM FSK
5 µM FSK
10 µM FSK
30 µM FSK

Figure S1. Viability after treatment with inhibitors used for blocking GPR55-related effectors and forskolin (FSK) in *GBM #4* and *GBM #10*. *GBM #4* and *GBM #10* were untreated (CTL) or exposed to Y-27632, U73122, U73343, 2-APB, PTX, gallein, CsA or FK506 (a) as well as to increasing concentration of FSK (b) for 24 h. Mitochondrial activity reflecting cellular viability were not affected in *GBM #4 and GBM #10* after treatment. Data are means ± SEM of N= 3-6 independent experiments performed with six technical replica and were normalized to the untreated control group. Significance was chosen for $p < 0.05$.

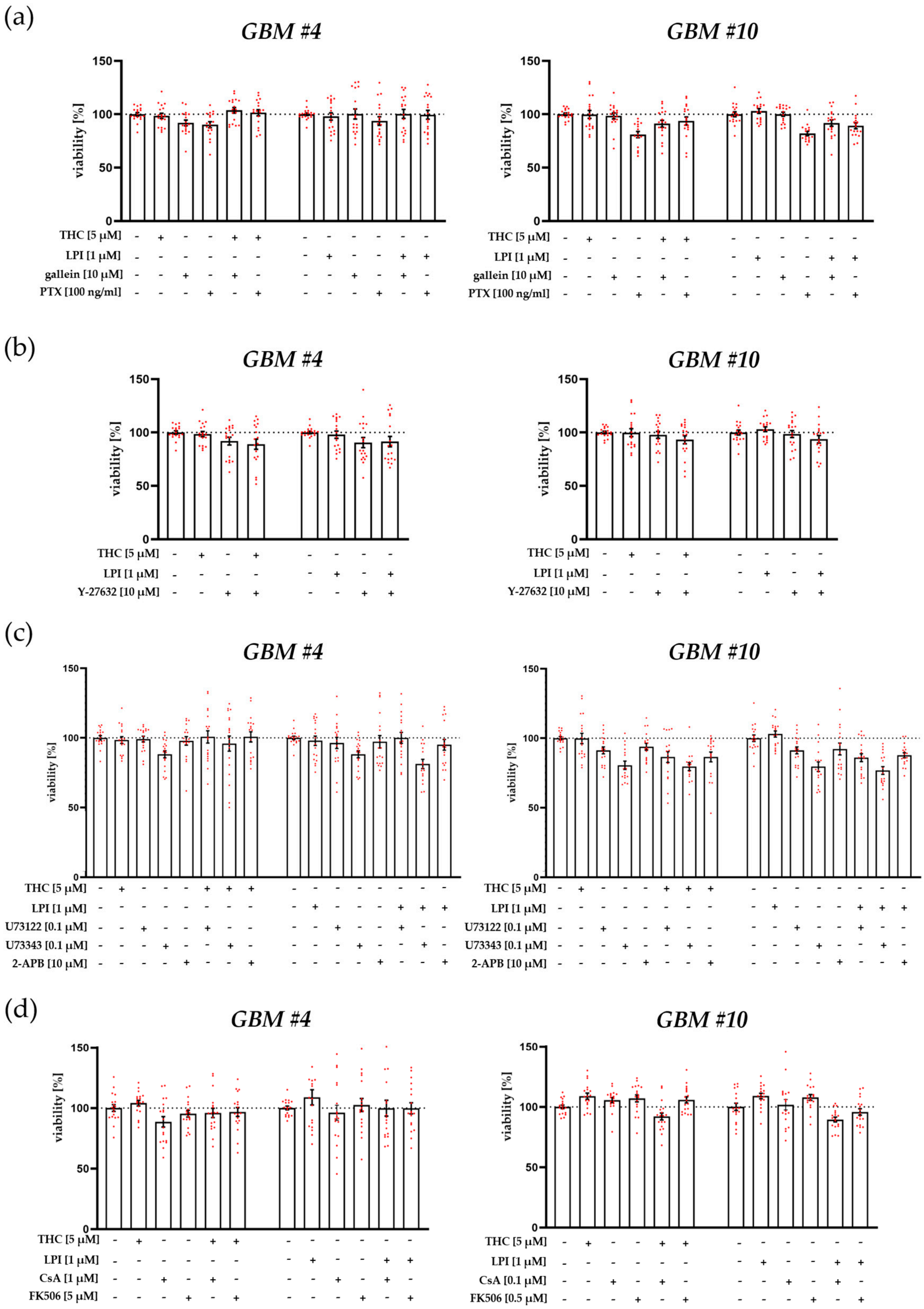

Figure S2. Viability after treatment with inhibitors used for blocking GPR55-related effectors in presence of THC or LPI in *GBM #4* and *GBM #10*. *GBM #4* and *GBM #10* were left untreated or exposed to PTX or gallein (a), Y-27632 (b), U73122, U73343 or 2-APB (c) and CsA or FK506 (d). Mitochondrial activity reflecting cellular viability was not affected in *GBM #4 and GBM #10* after co-treatment of inhibitors with THC or LPI. Data are means ± SEM of N=3 independent experiments performed with six technical replica and were normalized to the untreated control group. Significance was chosen for $p< 0.05$.

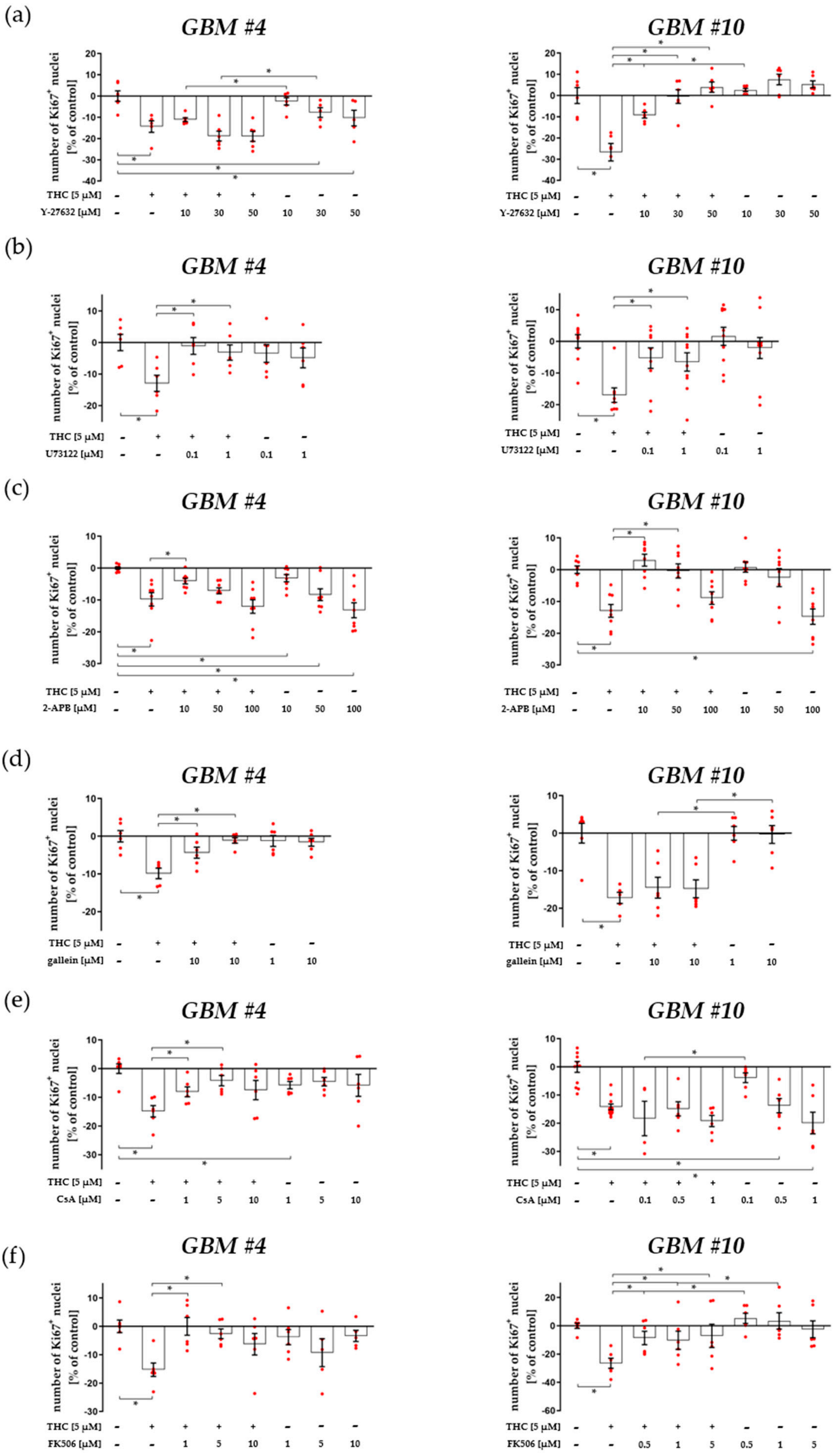
(a)
GBM #4
GBM #10
number of Ki67+ nuclei [% of control]
THC [5 µM]
Y-27632 [µM]
(b)
GBM #4
GBM #10
number of Ki67+ nuclei [% of control]
THC [5 µM]
U73122 [µM]
(c)
GBM #4
GBM #10
number of Ki67+ nuclei [% of control]
THC [5 µM]
2-APB [µM]
(d)
GBM #4
GBM #10
number of Ki67+ nuclei [% of control]
THC [5 µM]
gallein [µM]
(e)
GBM #4
GBM #10
number of Ki67+ nuclei [% of control]
THC [5 µM]
CsA [µM]
(f)
GBM #4
GBM #10
number of Ki67+ nuclei [% of control]
THC [5 µM]
FK506 [µM]

Figure S3. Impact of increasing concentrations of different inhibitors on THC-induced reduction of the number of Ki67⁺ nuclei. *GBM #4* and *GBM #10* were left untreated or exposed to THC for 24 h (a-f), resulting in a decreased number of $Ki67^+$ nuclei. In *GBM #4*, THC effects remained unaffected by Y-27632 (a) and were significantly reduced by U73122 (b), 2-APB (c), gallein (d), CsA (e) and FK506 (f). In *GBM #10* THC effects were abolished by Y-27632 (a), U73122 (b), 2-APB (c) and FK506 (d), but not by gallein (d) and CsA (e). Data are means ± SEM of N= 3 independent experiments performed in duplicate. Significance was chosen for $p < 0.05$. The asterisk denotes significant results regarding the respective measurement indicated with the bar.

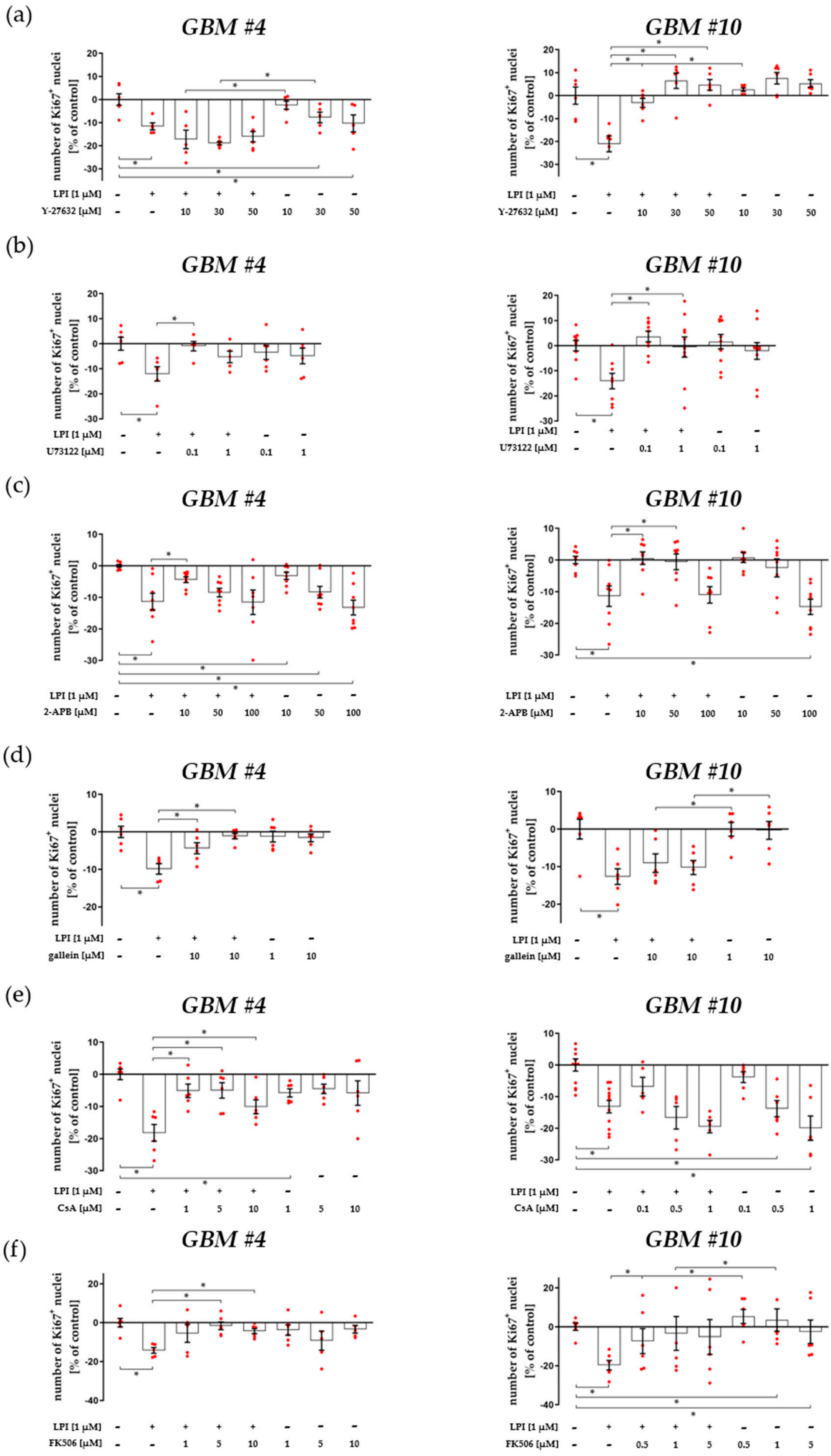
(a)
GBM #4
GBM #10
number of Ki67+ nuclei [% of control]
LPI [1 µM]
Y-27632 [µM]
10 30 50 10 30 50
(b)
GBM #4
GBM #10
number of Ki67+ nuclei [% of control]
LPI [1 µM]
U73122 [µM]
0.1 1 0.1 1
(c)
GBM #4
GBM #10
number of Ki67+ nuclei [% of control]
LPI [1 µM]
2-APB [µM]
10 50 100 10 50 100
(d)
GBM #4
GBM #10
number of Ki67+ nuclei [% of control]
LPI [1 µM]
gallein [µM]
10 10 1 10
(e)
GBM #4
GBM #10
number of Ki67+ nuclei [% of control]
LPI [1 µM]
CsA [µM]
1 5 10 1 5 10
0.1 0.5 1 0.1 0.5 1
(f)
GBM #4
GBM #10
number of Ki67+ nuclei [% of control]
LPI [1 µM]
FK506 [µM]
1 5 10 1 5 10
0.5 1 5 0.5 1 5

Figure S4. Impact of increasing concentrations of different inhibitors on THC-induced reduction of the number of Ki67⁺ nuclei. *GBM #4* and *GBM #10* were left untreated or exposed to LPI for 24 h (a-f), resulting in a decreased number of Ki67⁺ nuclei. In *GBM #4*, LPI effects remained unaffected by Y-27632 (a) and were significantly reduced by U73122 (b), 2-APB (c), gallein (d), CsA (e) and FK506 (f). In *GBM #10* LPI effects were abolished by Y-27632 (a), U73122 (b), 2-APB (c) and FK506 (d), but not by gallein (d) and CsA (e). Data are means ± SEM of N= 3 independent experiments performed in duplicate. Significance was chosen for $p < 0.05$. The asterisk denotes significant results regarding the respective measurement indicated with the bar.

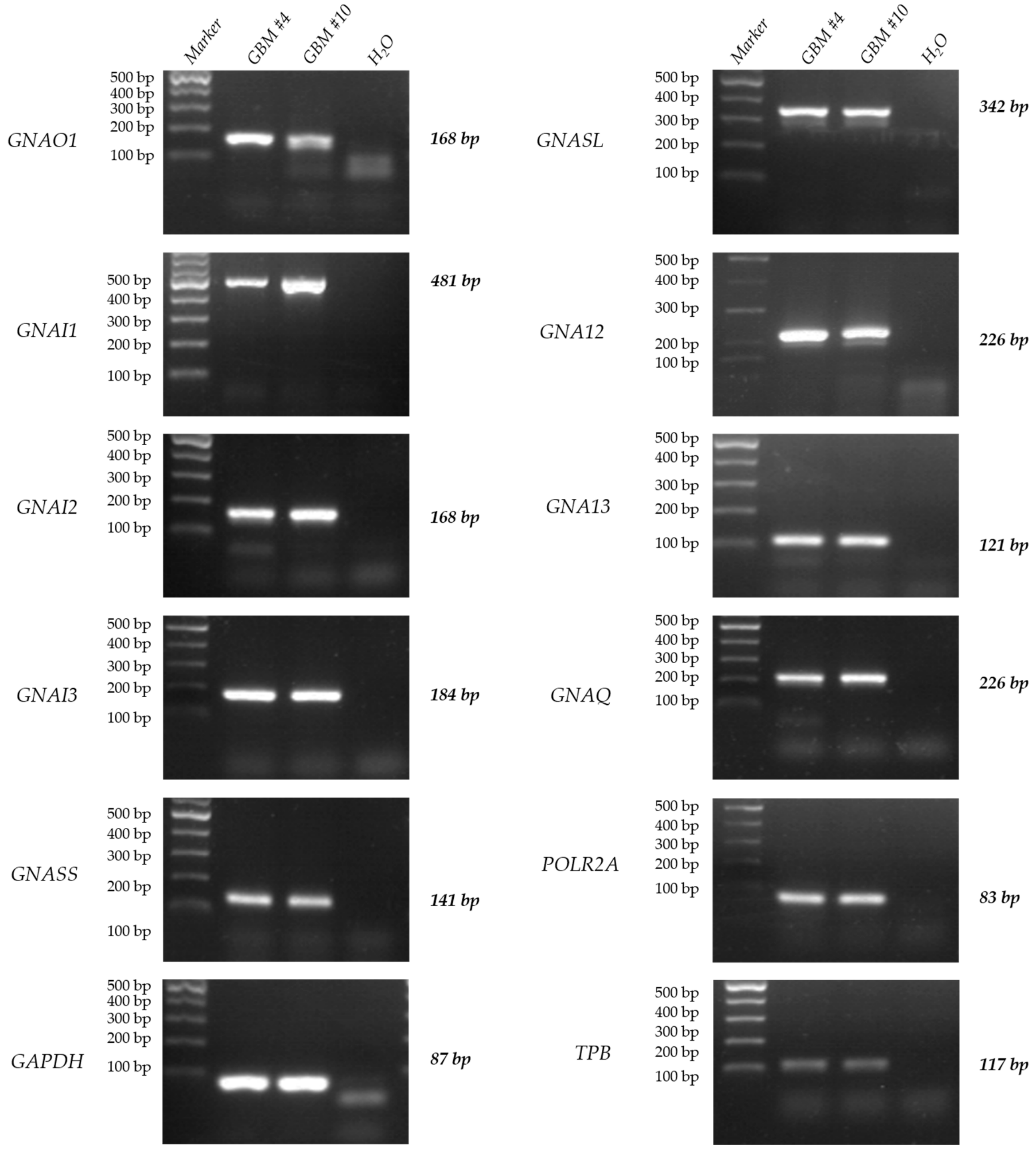

Figure S5. Original images of PCR products of different Gα subunits.

G$\alpha_{o/i}$-subunits

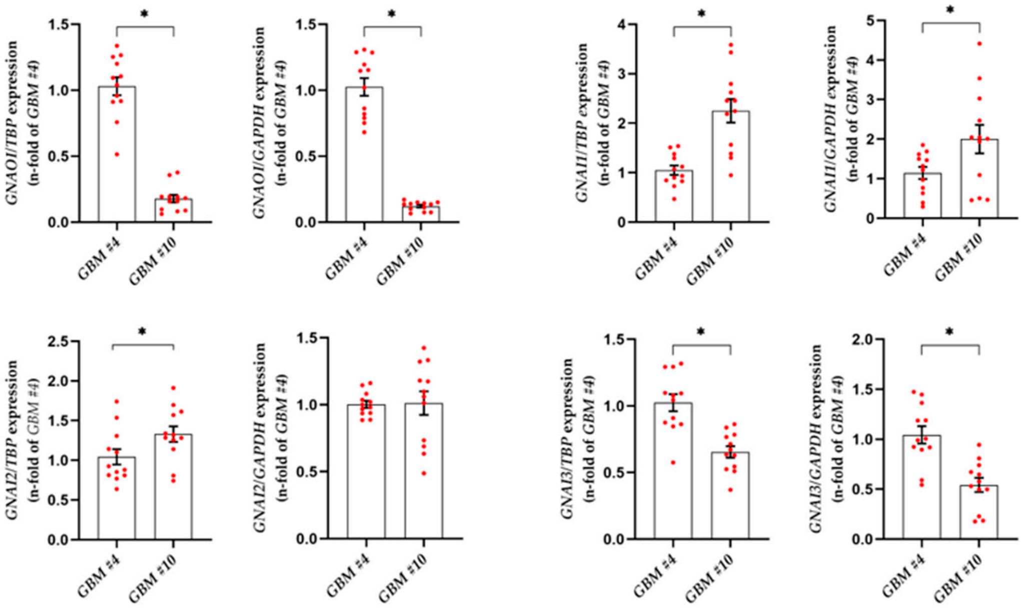

Gα_s-subunits

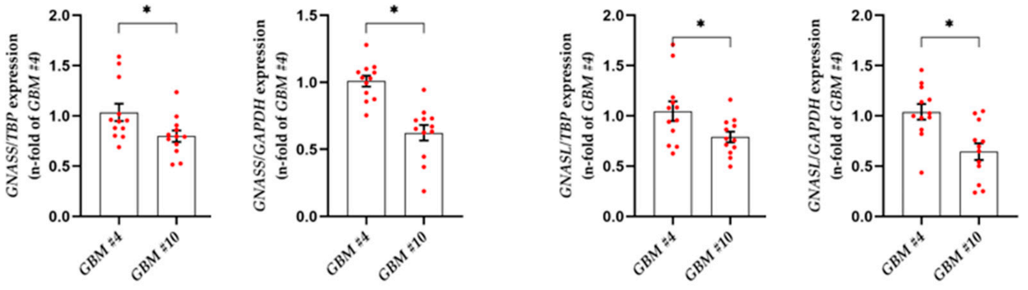

G$\alpha_{12/13}$-subunits

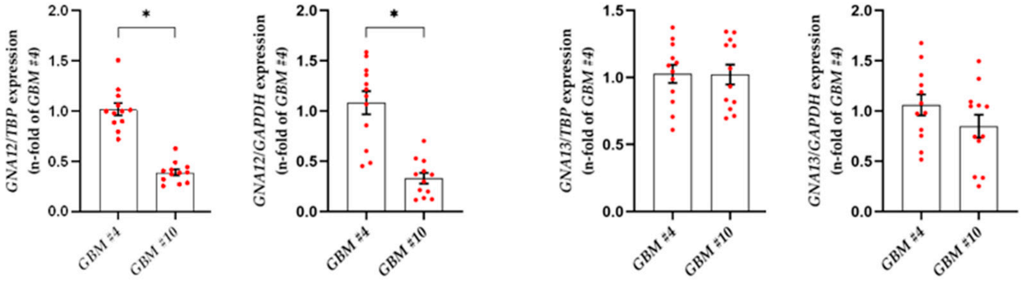

Gα_q-subunit

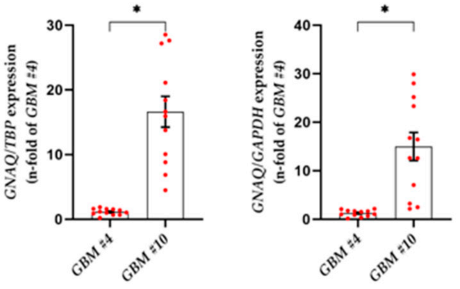

Figure S6. Quantification of genes encoding different Gα-subunits at transcript level using different housekeeping genes. Expression of *GNAO1*, *GNAI1*, *GNAI2*, *GNAI3*, *GNASS*, *GNASL*, *GNA12*, *GNA13*, and *GNAQ* were

analyzed by quantitative RT-PCR in untreated cells of *GBM #4* and *GBM #10*. *TATA-binding protein* (TBP) and *glyceraldehyde 3-phosphate dehydrogenase* (GAPDH) served as internal reference. Relative transcript levels were calculated using $2^{-\Delta\Delta Ct}$ method. No differences in the results of quantified transcript levels after normalization to TPB or GAPDH compared to the normalization to POLR2A were evident. Remarkably, *GBM #4* showed significantly higher amount of $G\alpha_o$ transcripts than *GBM #10*, whereas $G\alpha_q$ was significant higher expressed by *GBM #10* when compared to *GBM #4*. Data represent means ± SEM (normalized to *GBM #4* or *GNAOI*) of N=4 independent experiments performed in triplicate. Significance was chosen for $p < 0.05$. The asterisk denotes significant results regarding the respective measurement indicated with the bar.

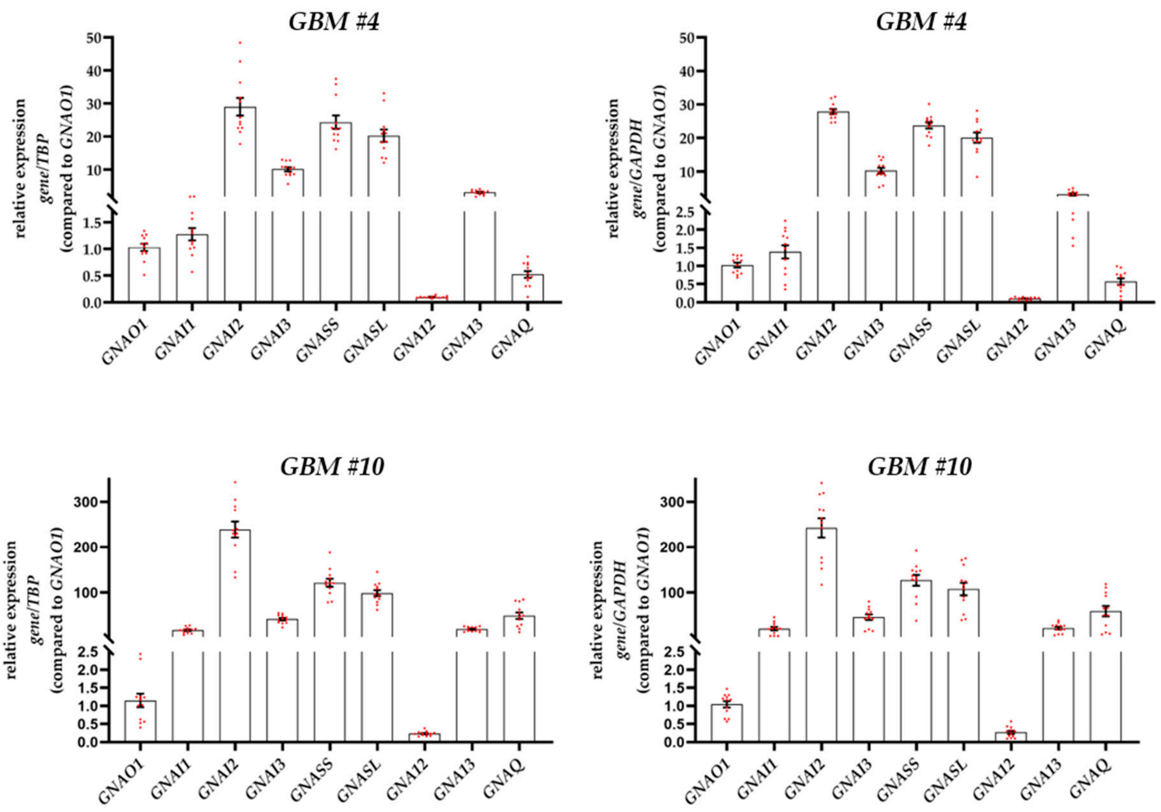

Figure S7. Abundance and distribution of genes encoding different Gα-subunits at transcript level using different housekeeping genes. Expression of *GNAO1, GNAI1, GNAI2, GNAI3, GNASS, GNASL, GNA12, GNA13*, and *GNAQ* were analyzed by quantitative RT-PCR in untreated cells of *GBM #4* and *GBM #10*. *TATA-binding protein* (TBP) and *glyceraldehyde 3-phosphate dehydrogenase* (GAPDH) served as internal reference. Relative transcript levels were calculated using $2^{-\Delta\Delta Ct}$ method. The abundance and distribution of gene transcripts encoding different subunits within one cell population were similar after normalization to TBP or GAPDH compared to the normalization to POLR2A. Data represent means ± SEM (normalized to *GBM #4* or *GNAOI*) of N=4 independent experiments performed in triplicate.

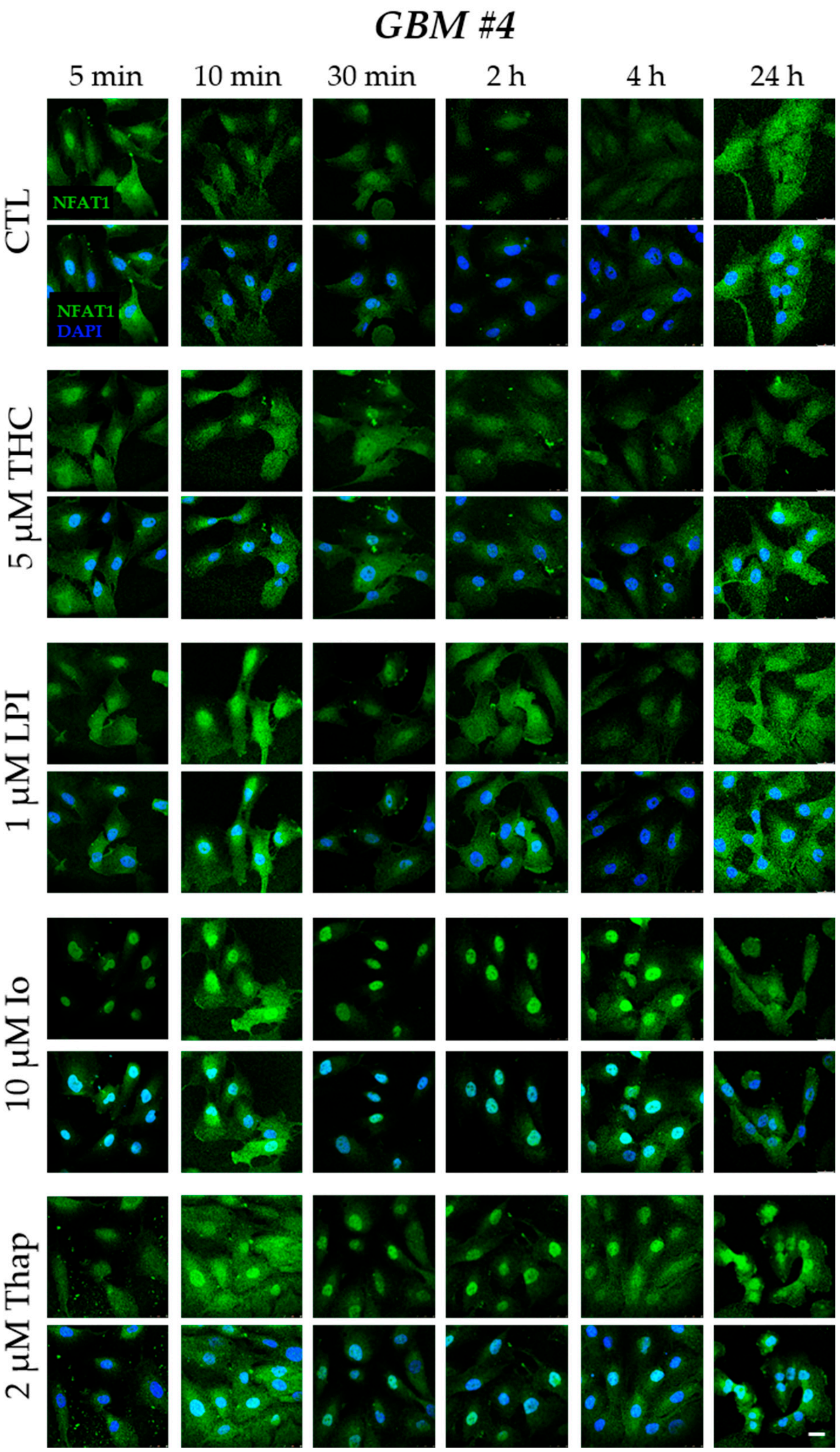

Figure S8. Localization of NFAT1 in *GBM #4* treated with THC, LPI, Io and Thap at different time points. Representative images of NFAT1 in untreated and THC (5 µM)-, LPI (1 µM)-, ionomycin (10 µM)- and thapsigargin (2 µM)-treated cells of *GBM #4* after 5 min, 10 min, 30 min, 2 h, 4 h, and 24 h. Resident NFAT1 is located in the cytoplasm, whereas active NFAT1 is located in the nucleus. Increased signals of NFAT1 in the nucleus were found in early time points compared with late in the untreated control groups after changing the medium. Altered nuclear signals were detected after Io (5 min) and Thap (10 min) treatment compared with the untreated control group. THC and LPI

had no visible effects. Cell nuclei were counterstained with DAPI. Scale bar = 25 μm.

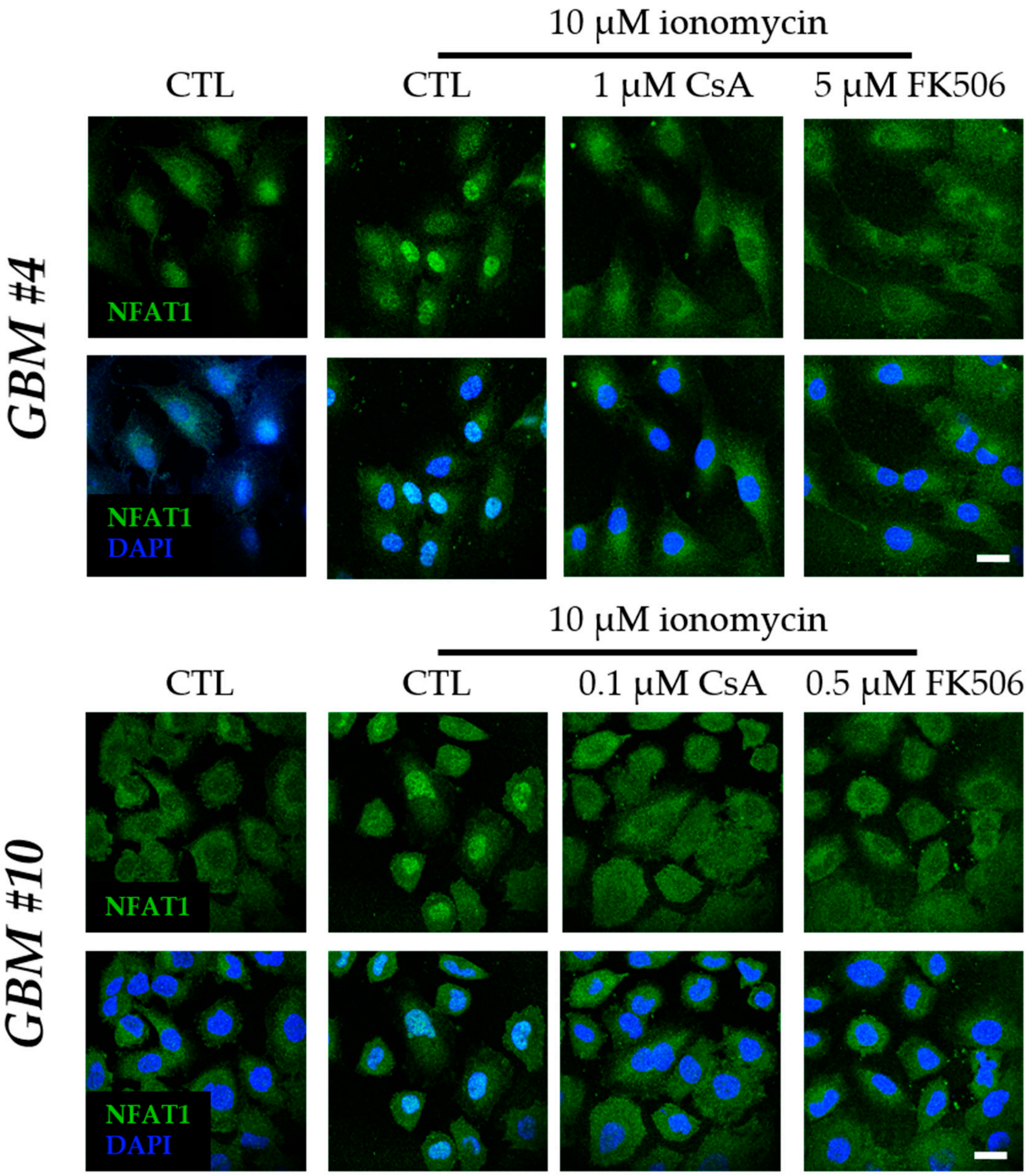

Figure S9: Influence of ionomycin on the subcellular localization of NFAT1 after 30 min in presence of CsA and FK506. Ionomycin (Io) induced a markedly translocation of NFAT1 into the nucleus after 30 min. In the presence of CsA and FK506, ionomycin effects were significantly abolished. Cell nuclei were counterstained with DAPI. Scale bar = 25 μm.

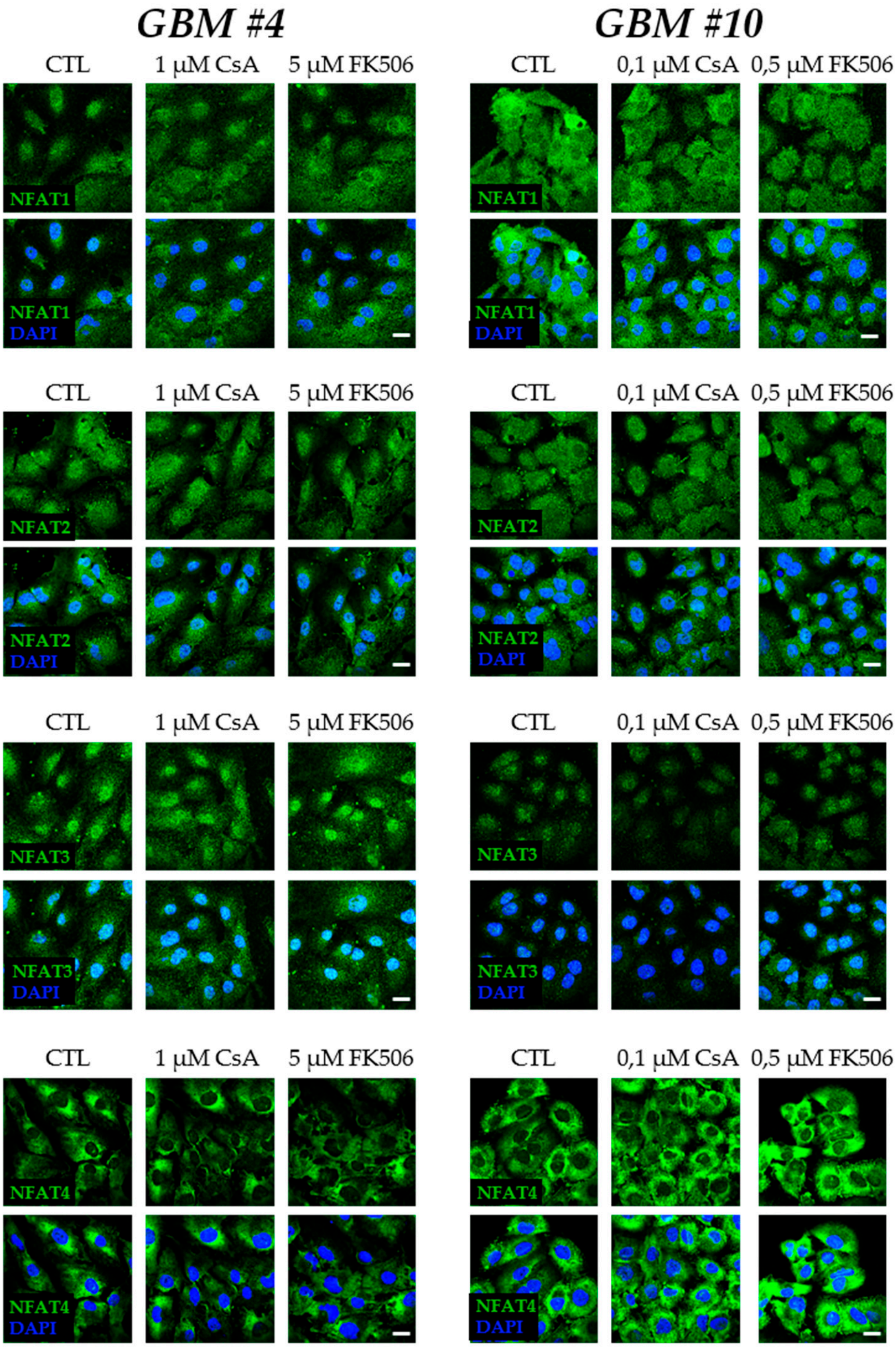

Figure S10: Subcellular localization of NFAT1-4 after treatment with FK506 and CsA. Representative images of NFAT1-4 in untreated and FK506 and CsA treated cells of *GBM #4* and *GBM #10* after 30 min. Cells were pre-incubated for 1 h with CsA or 1.5 h with FK506. For *GBM #4*, it was shown that NFAT1 was mainly present in the cytoplasm after CsA and FK506 stimulation, whereas nuclear signals were reduced and not evident when compared to the untreated control group. In contrast, nuclear signals of NFAT2 and NFAT3 remained unchanged after calcineurin inhibition by FK506 or CsA in both cell lines. Cell nuclei were counterstained with DAPI. Scale bar = 25 µm.

Table S2. Exact measurement values and sample sizes after treatment with THC and LPI in presence of Y-27632.

cell type	treatment	mean [%]	SEM [%]	sample size
GBM #4	CTL	0.00	2.48	6
	THC	-14.36	2.72	6
	LPI	-11.64	1.51	5
	10 μM Y-27632	-2.48	1.72	6
	30 μM Y27632	-7.77	2.29	5
	50 μM -27632	-10.36	3.71	5
	THC + 10 μM Y-27632	-11.15	0.93	6
	THC + 30 μM Y-27632	-18.84	2.31	6
	THC + 50 μM Y-27632	-18.96	2.37	6
	LPI + 10 μM Y-27632	-17.27	4.00	5
	LPI + 30 μM Y-27632	-18.97	0.69	6
	LPI + 50 μM Y-27632	-16.09	2.25	6
GBM #10	CTL	0.00	3.76	6
	THC	-26.73	4.11	6
	LPI	-21.03	3.44	6
	10 μM Y-27632	2.63	0.75	6
	30 μM Y27632	7.57	2.51	6
	50 μM -27632	5.29	1.63	6
	THC + 10 μM Y-27632	-9.26	1.39	6
	THC + 30 μM Y-27632	-0.46	3.24	6
	THC + 50 μM Y-27632	3.96	2.44	6
	LPI + 10 μM Y-27632	-3.17	1.75	6
	LPI + 30 μM Y-27632	6.48	3.38	6
	LPI + 50 μM Y-27632	4.67	2.31	6

Table S3. Exact measurement values and sample sizes after treatment with THC and LPI in presence of U73122.

cell type	treatment	mean [%]	SEM [%]	sample size
GBM #4	CTL	0.00	2.59	6
	THC	-12.97	2.56	6
	LPI	-12.08	2.85	6
	0.1 μM U73122	-3.50	2.81	6
	1 μM U73122	-4.89	3.16	6
	THC + 0.1 μM U73122	-1.10	2.70	6
	THC + 1 μM U73122	-3.18	2.44	6
	LPI + 0.1 μM U73122	-1.01	1.88	5
	LPI + 1 μM U73122	-5.28	2.32	5
GBM #10	CTL	0.00	2.12	9
	THC	-17.04	2.28	8
	LPI	-16.42	3.56	9
	0.1 μM U73122	1.60	2.87	10
	1 μM U73122	-2.12	3.33	10
	THC + 0.1 μM U73122	-5.30	3.26	9
	THC + 1 μM U73122	-6.53	2.91	10
	LPI + 0.1 μM U73122	3.65	2.12	9
	LPI + 1 μM U73122	-0.54	4.06	10

Table S4. Exact measurement values and sample sizes after treatment with THC and LPI in presence of U73343.

cell type	treatment	mean [%]	SEM [%]	sample size
GBM #4	CTL	0.00	1.01	6
	THC	-17.09	3.63	6
	LPI	-15.70	3.85	6
	U73433	-1.36	1.76	6
	THC + U73433	-13.14	1.61	6
	LPI + U73433	-14.51	2.98	5
GBM #10	CTL	0.00	1.25	5
	THC	-19.79	4.30	6
	LPI	-19.04	3.22	6
	U73433	-0.71	1.61	6
	THC + U73433	-21.90	4.65	5
	LPI + U73433	-15.32	1.82	6

Table S5. Exact measurement values and sample sizes after treatment with THC and LPI in presence of 2-APB.

cell type	gene	mean [%]	SEM [%]	sample size
GBM #4	CTL	0.00	0.38	8
	THC	-9.79	2.12	8
	LPI	-11.33	2.66	8
	10 μM 2-APB	-3.16	1.15	8
	50 μM 2-APB	-8.34	1.85	8
	100 μM 2-ABP	-13.25	2.36	8
	THC + 10 μM 2-APB	-4.06	0.90	8
	THC + 50 μM 2-APB	-7.13	0.96	8
	THC + 100 μM 2-APB	-12.06	2.12	8
	LPI + 10 μM 2-APB	-4.38	0.92	8
	LPI + 50 μM 2-APB	-8.47	1.36	8
	LPI + 100 μM 2-APB	-11.55	3.89	7
GBM #10	CTL	0.00	1.18	8
	THC	-12.96	2.01	8
	LPI	-11.39	3.24	8
	10 μM 2-APB	0.82	1.59	8
	50 μM 2-APB	-2.48	2.84	8
	100 μM 2-ABP	-14.78	2.43	8
	THC + 10 μM 2-APB	3.01	1.89	8
	THC + 50 μM 2-APB	-0.39	2.22	8
	THC + 100 μM 2-APB	-8.93	1.99	8
	LPI + 10 μM 2-APB	0.58	1.98	8
	LPI + 50 μM 2-APB	-0.56	2.50	8
	LPI + 100 μM 2-APB	-10.99	2.61	8

Table S6. Exact measurement values and sample sizes of the expression of different Gα subunits on transcript level measured by qRT-PCR. Data are normalized to the expression of certain genes in *GBM #4*.

gene	cell type	mean [%]	SEM [%]	sample size
GNAO1	*GBM #4*	1.02	0.06	12
GNAO1	*GBM #10*	0.15	0.02	12
GNAI1	*GBM #4*	1.10	0.12	12
GNAI1	*GBM #10*	2.04	0.25	12
GNAI2	*GBM #4*	1.01	0.03	12
GNAI2	*GBM #10*	1.13	0.05	12
GNAI3	*GBM #4*	1.02	0.06	12
GNAI3	*GBM #10*	0.58	0.04	12
GNASS	*GBM #4*	1.01	0.04	12
GNASS	*GBM #10*	0.69	0.03	12
GNASL	*GBM #4*	1.03	0.07	12
GNASL	*GBM #10*	0.69	0.04	12
GNA12	*GBM #4*	1.05	0.09	12
GNA12	*GBM #10*	0.35	0.04	12
GNA13	*GBM #4*	1.03	0.07	12
GNA13	*GBM #10*	0.90	0.07	12
GNAQ	*GBM #4*	1.19	0.16	12
GNAQ	*GBM #10*	14.96	2.12	12

Table S7. Exact measurement values and sample sizes of the expression of different Gα subunits on transcript level measured by qRT-PCR in *GBM #4* and *GBM #10*. Data are normalized to the expression of *GNAO1* in *GBM #4* and *GBM #10*, respectively.

cell type	gene	mean [%]	SEM [%]	sample size
GBM #4	*GNAO1*	1.02	0.06	12
	GNAI1	1.33	0.15	12
	GNAI2	27.94	0.85	12
	GNAI3	10.10	0.62	12
	GNASS	23.74	0.85	12
	GNASL	19.97	0.14	12
	GNA12	0.10	0.01	12
	GNA13	3.10	0.21	12
	GNAQ	0.55	0.07	12
GBM #10	*GNAO1*	1.08	0.12	12
	GNAI1	18.39	2.28	12
	GNAI2	233.6	10.15	12
	GNAI3	42.24	2.96	12
	GNASS	119.8	5.92	12
	GNASL	98.86	6.22	12
	GNA12	0.25	0.03	12
	GNA13	20.10	1.59	12
	GNAQ	51.12	7.24	12

Table S8. Exact measurement values and sample sizes after treatment with THC and LPI in presence of PTX.

cell type	treatment	mean [%]	SEM [%]	sample size
GBM #4	CTL	0.00	1.29	6
	THC	-6.72	1.74	5
	PTX	-8.41	1.88	6
	THC + PTX	-14.71	3.27	6
	CTL	0.00	3.36	6
	LPI	-12.48	3.81	6
	PTX	-22.54	3.41	6
	LPI + PTX	-22.54	3.80	6
GBM #10	CTL	0.00	1.96	6
	THC	-20.49	4.64	6
	PTX	-15.63	3.27	6
	THC + PTX	-23.09	3.40	6
	CTL	0.00	1.96	6
	LPI	-14.97	4.32	6
	PTX	-15.63	3.27	6
	LPI + PTX	-18.77	2.31	6

Table S9. Exact measurement values and sample sizes after treatment with increasing FSK concentrations.

cell type	treatment	mean [%]	SEM [%]	sample size
GBM #4	CTL	0.00	0.79	6
	0.1 µM FSK	-5.39	2.03	6
	1 µM FSK	-10.15	2.55	6
	5 µM FSK	-13.42	1.79	6
	10 µM FSK	-16.74	2.18	6
	30 µM FSK	-11.86	0.87	6
GBM #10	CTL	0.00	1.73	6
	0.1 µM FSK	-3.46	1.93	6
	1 µM FSK	-8.34	1.78	6
	5 µM FSK	-8.01	2.66	6
	10 µM FSK	-7.93	3.63	6
	30 µM FSK	-11.21	2.57	6

Table S10. Exact measurement values and sample sizes after treatment with THC and LPI in presence of Gallein.

cell type	treatment	mean [%]	SEM [%]	sample size
GBM #4	CTL	0.00	1.51	6
	THC	-11.33	1.45	6
	LPI	-9.87	1.40	5
	1 µM gallein	-1.25	1.44	6
	10 µM gallein	-1.58	1.05	6
	THC + 1 µM gallein	-5.61	0.97	6
	THC + 10 µM gallein	-0.68	0.88	6
	LPI + 1 µM gallein	-4.37	1.47	6
	LPI + 10 µM gallein	-1.12	0.72	6
GBM #10	CTL	0.00	2.65	6
	THC	-17.26	1.49	5
	LPI	-12.68	2.10	6
	1 µM gallein	-0.04	1.83	6
	10 µM gallein	-0.34	2.37	6
	THC + 1 µM gallein	-14.55	2.78	6
	THC + 10 µM gallein	-14.84	2.39	6
	LPI + 1 µM gallein	-9.09	2.47	6
	LPI + 10 µM gallein	-10.26	1.87	6

Table S11. Exact measurement values and sample sizes after treatment with THC and LPI in presence of CsA.

cell type	treatment	mean [%]	SEM [%]	sample size
GBM #4	CTL	0.00	1.67	6
	THC	-14.89	2.00	6
	LPI	-18.23	2.59	6
	1 µM CsA	-5.77	1.27	6
	5 µM CsA	-4.54	1.47	6
	10 µM CsA	-5.84	3.82	6
	THC + 1 µM CsA	-8.09	1.73	6
	THC + 5 µM CsA	-4.18	1.81	6
	THC + 10 µM CsA	-7.47	3.37	6
	LPI + 1 µM CsA	-5.16	2.11	6
	LPI + 5 µM CsA	-5.02	2.40	6
	LPI + 10 µM CsA	-10.10	2.15	6
GBM #10	CTL	0.00	1.93	12
	THC	-14.23	1.04	11
	LPI	-13.17	1.97	11
	0.1 µM CsA	-3.86	1.71	6
	0.5 µM CsA	-13.79	2.55	6
	1 µM CsA	-19.96	3.83	6
	THC + 0.1 µM CsA	-18.34	6.13	4
	THC + 0.5 µM CsA	-14.91	2.52	6
	THC + 1 µM CsA	-19.23	1.98	6
	LPI + 0.1 µM CsA	-6.86	2.98	5
	LPI + 0.5 µM CsA	-14.91	2.52	6
	LPI + 1 µM CsA	-19.23	1.98	6

Table S12. Exact measurement values and sample sizes after treatment with THC and LPI in presence of FK506.

cell type	treatment	mean [%]	SEM [%]	sample size
GBM #4	CTL	0.00	2.20	6
	THC	-15.28	2.37	6
	LPI	-17.72	3.66	6
	1 μM FK506	-3.80	2.67	6
	5 μM FK506	-9.27	4.91	5
	10 μM FK506	-3.38	1.99	5
	THC + 1 μM FK506	0.00	3.13	6
	THC + 5 μM FK506	-2.61	1.73	6
	THC + 10 μM FK506	-6.29	3.79	6
	LPI + 1 μM FK506	-5.48	4.63	5
	LPI + 5 μM FK506	-1.61	2.00	6
	LPI + 10 μM FK506	-4.38	1.29	6
GBM #10	CTL	0.00	1.84	6
	THC	-26.52	3.54	6
	LPI	-18.46	2.68	6
	0.5 μM FK506	2.14	3.84	6
	1 μM FK506	-2.44	2.31	6
	5 μM FK506	-10.67	3.04	6
	THC + 0.5 μM FK506	-10.49	4.15	6
	THC + 1 μM FK506	-13.54	2.31	6
	THC + 5 μM FK506	-12.61	6.72	6
	LPI + 0.5 μM FK506	-10.37	2.20	6
	LPI + 1 μM FK506	-12.96	2.23	6
	LPI + 5 μM FK506	-14.52	2.98	6
